# Supplementary material for: Spatiotemporal patterns of the COVID-19 epidemic in Mexico at the municipality level
Source: PeerJ. 2021 Dec 24;9:e12685. doi: 10.7717/peerj.12685 (PMC8711283; doi:10.7717/peerj.12685)
Supplement: Supplemental Information 1 — Maps of weekly new cases number and clusters with associated relative risk [file peerj-09-12685-s001.pdf]

Spatiotemporal patterns of the COVID-19 pandemic in Mexico at the municipality level Supplementary Material

J.F. Mas

These Supplementary Materials contain maps of population density, weekly new cases number and clusters with associated their relative risk.

Contents

1 Map of population density 2

2 Maps of weekly new cases and clusters 3

3 Effect of the different types of distance on neighbouring relationships 7

4 AUCs obtained by the different types of distance 12

5 AUCs obtained by the different types of distance over time 15

6 Equivalence between week number and dates 19

1 Map of population density

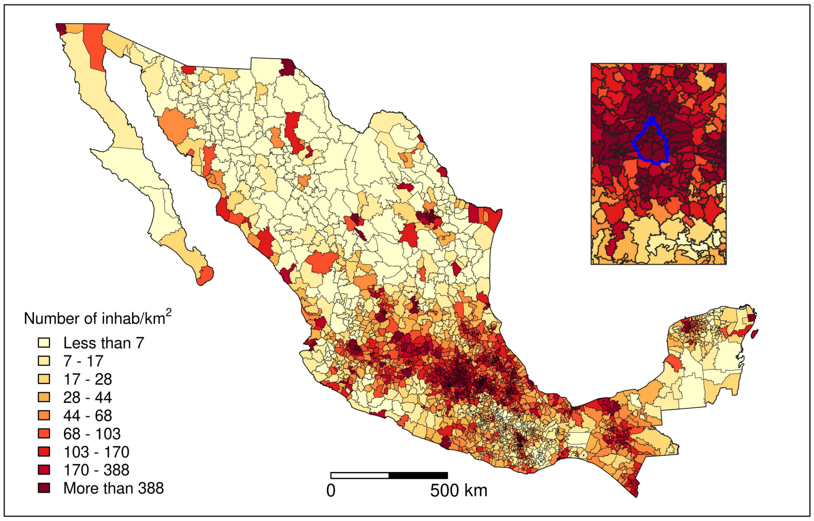

2 Maps of weekly new cases and clusters

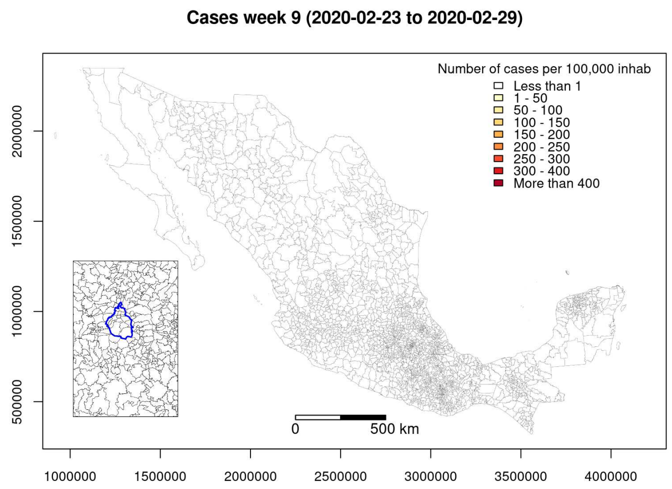

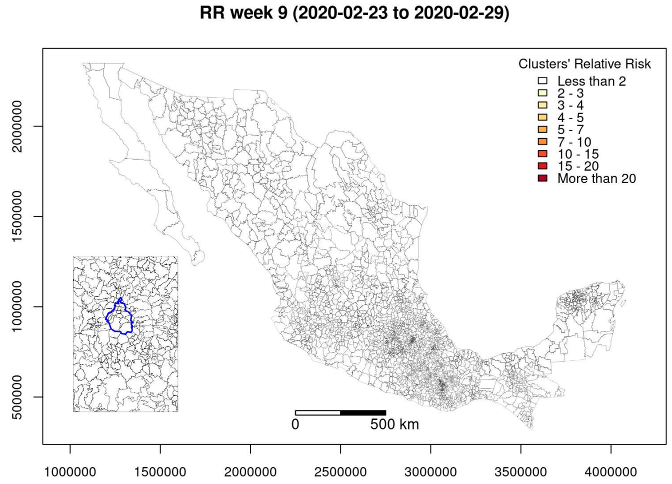

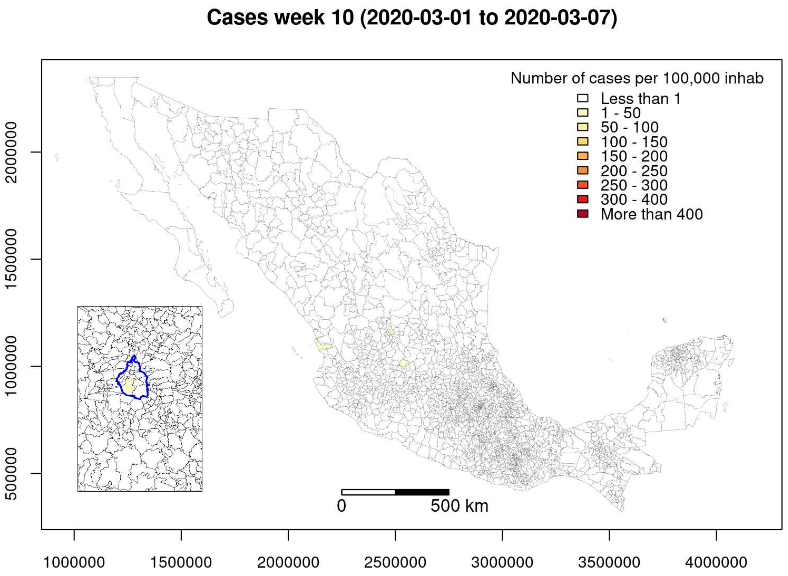
Cases week 10 (2020-03-01 to 2020-03-07)
Number of cases per 100,000 inhab
Less than 1
1 - 50
50 - 100
100 - 150
150 - 200
200 - 250
250 - 300
300 - 400
More than 400
0
500 km
2000000
1500000
1000000
500000
1000000
1500000
2000000
2500000
3000000
3500000
4000000

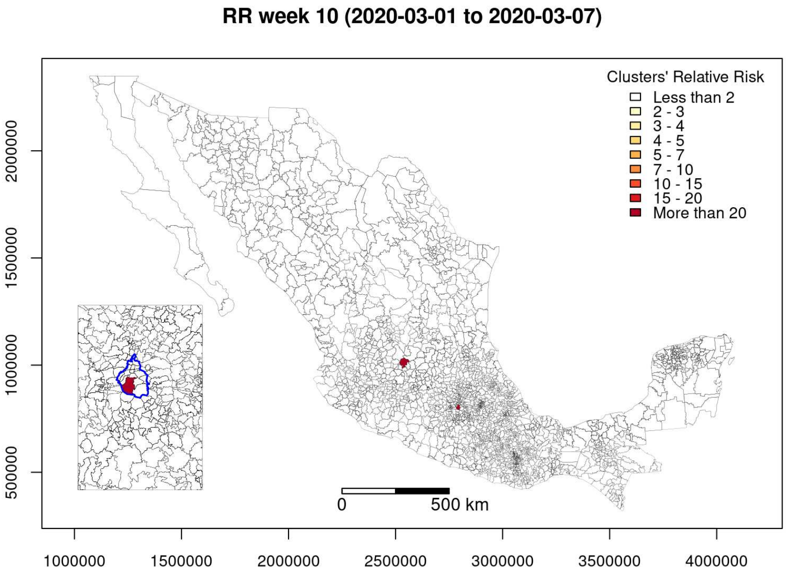
RR week 10 (2020-03-01 to 2020-03-07)
Clusters' Relative Risk
Less than 2
2 - 3
3 - 4
4 - 5
5 - 7
7 - 10
10 - 15
15 - 20
More than 20
0
500 km
2000000
1500000
1000000
500000
1000000
1500000
2000000
2500000
3000000
3500000
4000000

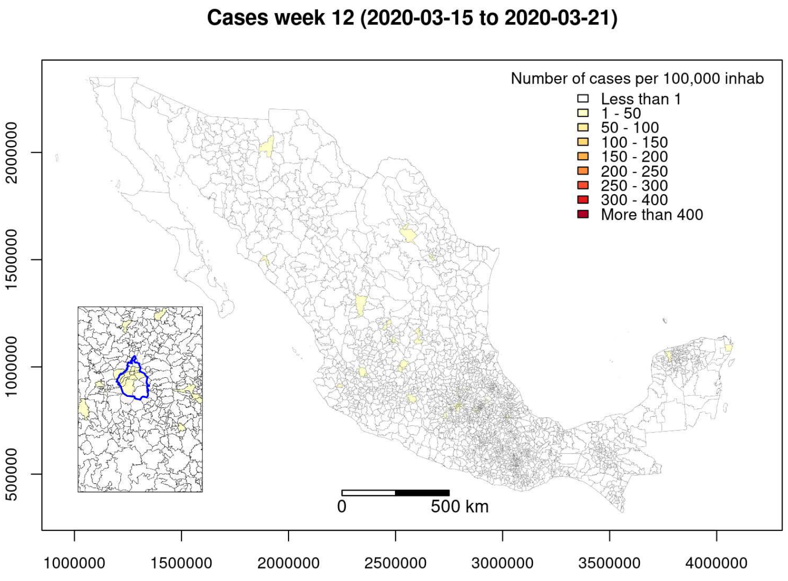
Cases week 12 (2020-03-15 to 2020-03-21)
Number of cases per 100,000 inhab
Less than 1
1 - 50
50 - 100
100 - 150
150 - 200
200 - 250
250 - 300
300 - 400
More than 400
0
500 km
500000
1000000
1500000
2000000
1000000
1500000
2000000
2500000
3000000
3500000
4000000

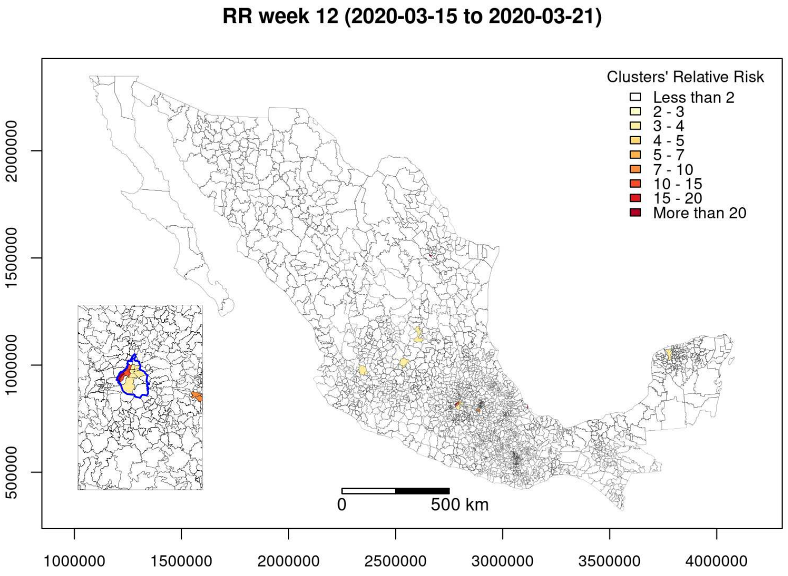
RR week 12 (2020-03-15 to 2020-03-21)
Clusters' Relative Risk
Less than 2
2 - 3
3 - 4
4 - 5
5 - 7
7 - 10
10 - 15
15 - 20
More than 20
0
500 km
500000
1000000
1500000
2000000
1000000
1500000
2000000
2500000
3000000
3500000
4000000

Cases week 14 (2020-03-29 to 2020-04-04)

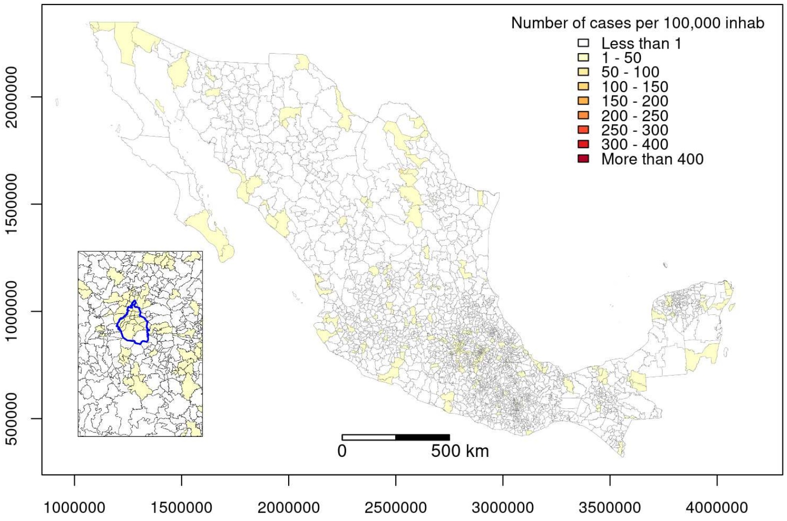

RR week 14 (2020-03-29 to 2020-04-04)

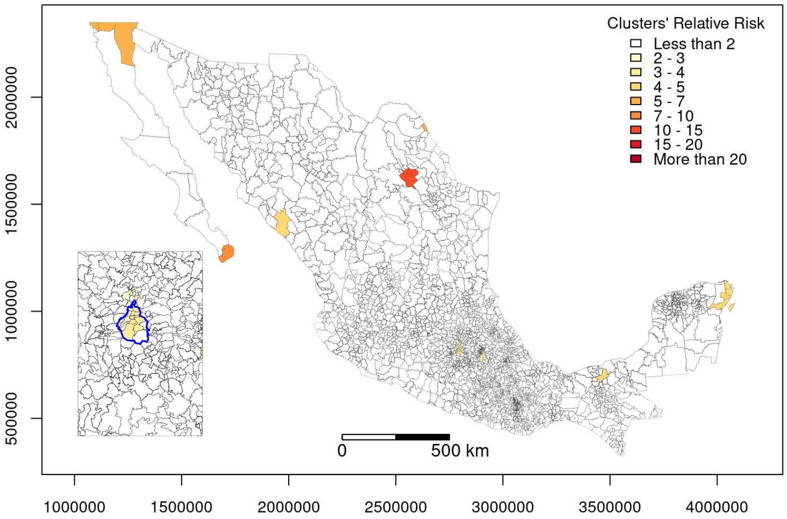

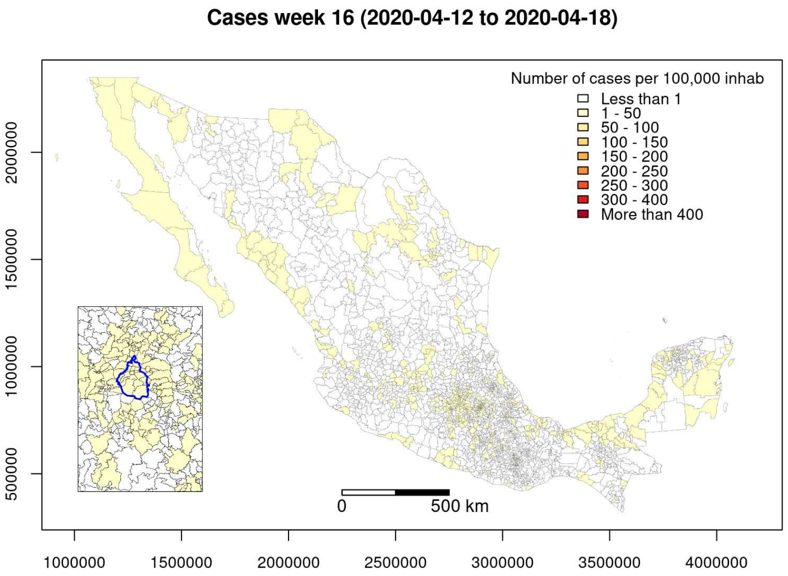
Cases week 16 (2020-04-12 to 2020-04-18)
Number of cases per 100,000 inhab
Less than 1
1 - 50
50 - 100
100 - 150
150 - 200
200 - 250
250 - 300
300 - 400
More than 400
0
500 km
500000
1000000
1500000
2000000
1000000
1500000
2000000
2500000
3000000
3500000
4000000

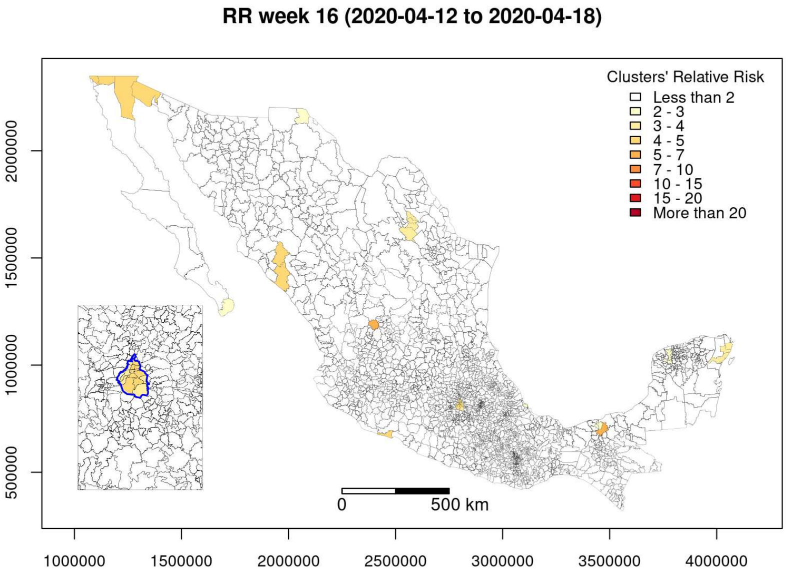
RR week 16 (2020-04-12 to 2020-04-18)
Clusters' Relative Risk
Less than 2
2 - 3
3 - 4
4 - 5
5 - 7
7 - 10
10 - 15
15 - 20
More than 20
0
500 km
500000
1000000
1500000
2000000
1000000
1500000
2000000
2500000
3000000
3500000
4000000

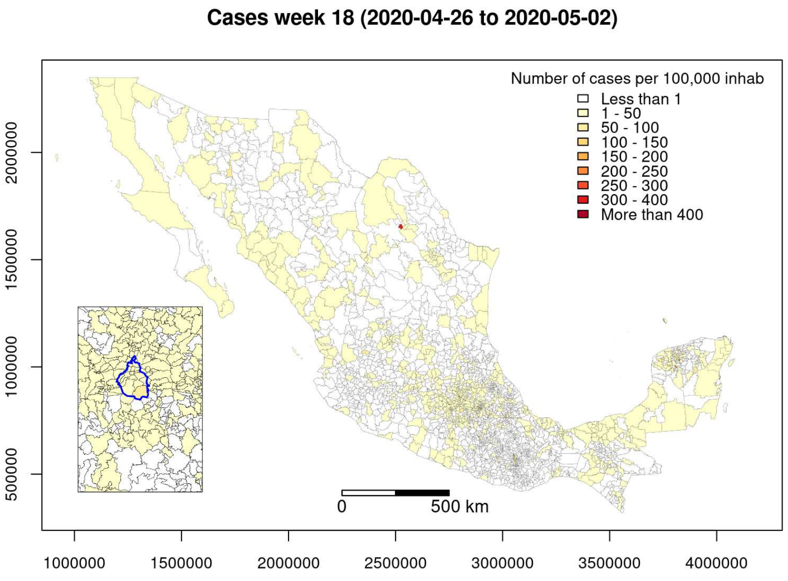
Cases week 18 (2020-04-26 to 2020-05-02)
Number of cases per 100,000 inhab
Less than 1
1 - 50
50 - 100
100 - 150
150 - 200
200 - 250
250 - 300
300 - 400
More than 400
0
500 km
500000
1000000
1500000
2000000
1000000
1500000
2000000
2500000
3000000
3500000
4000000

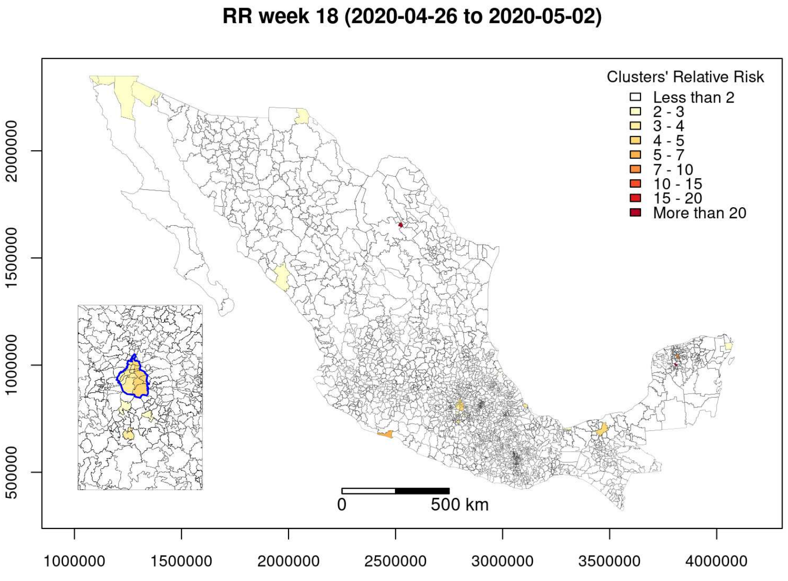
RR week 18 (2020-04-26 to 2020-05-02)
Clusters' Relative Risk
Less than 2
2 - 3
3 - 4
4 - 5
5 - 7
7 - 10
10 - 15
15 - 20
More than 20
0
500 km
500000
1000000
1500000
2000000
1000000
1500000
2000000
2500000
3000000
3500000
4000000

Cases week 20 (2020-05-10 to 2020-05-16)

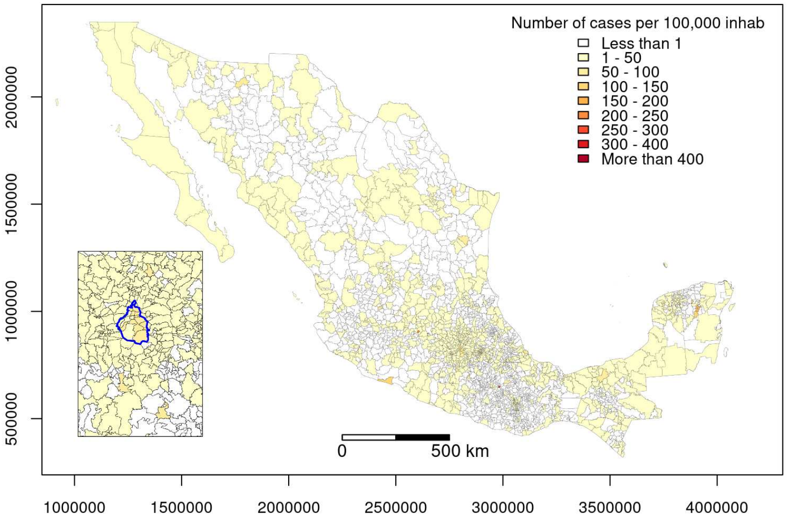

RR week 20 (2020-05-10 to 2020-05-16)

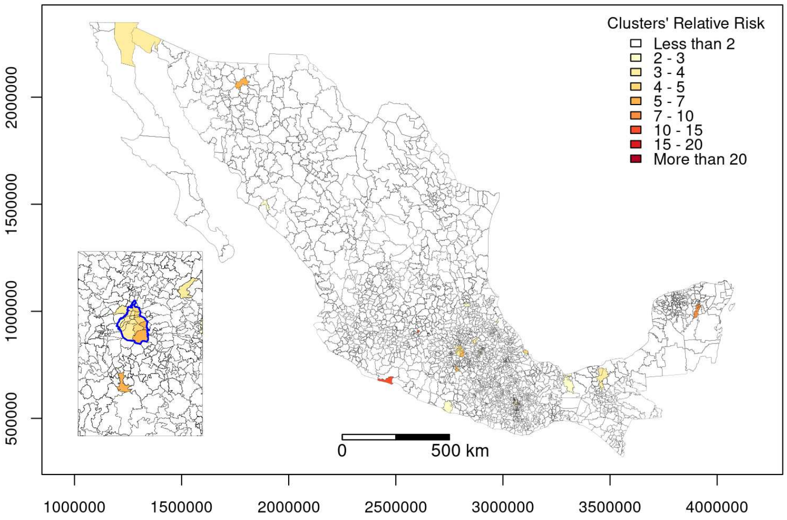

Cases week 22 (2020-05-24 to 2020-05-30)

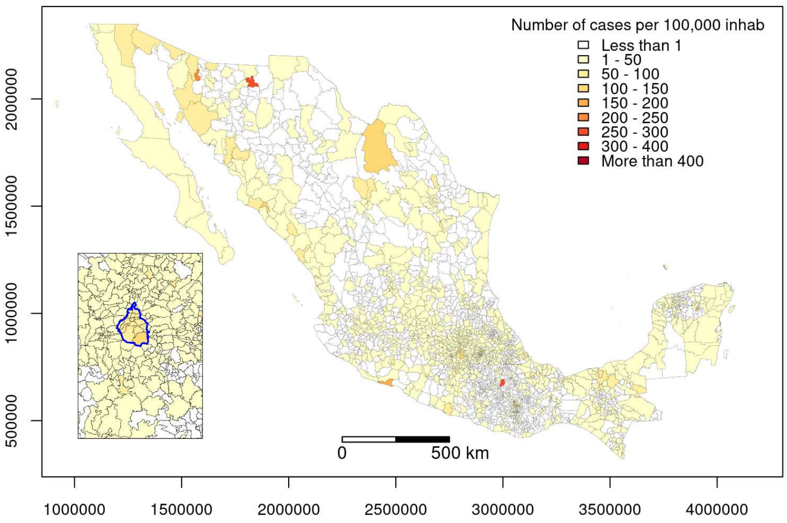

RR week 22 (2020-05-24 to 2020-05-30)

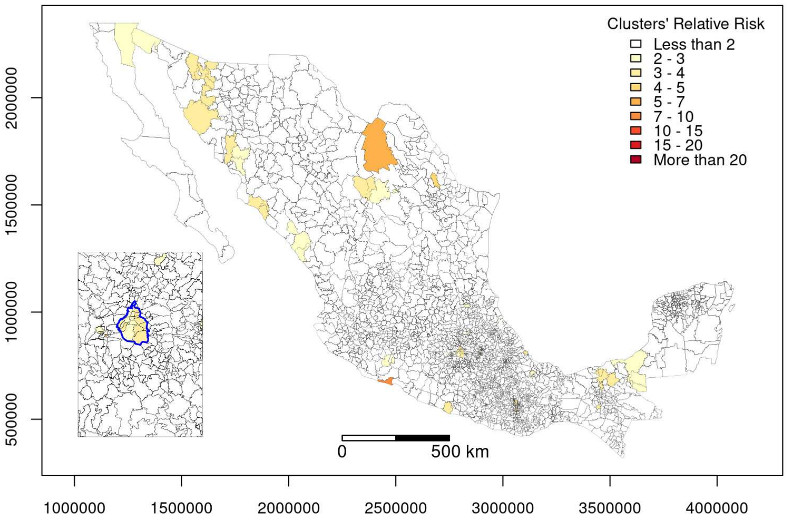

Cases week 24 (2020-06-07 to 2020-06-13)

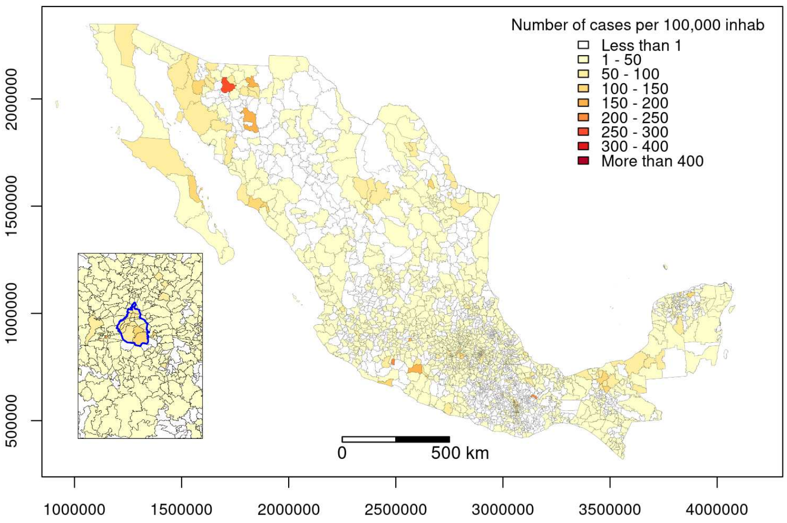

RR week 24 (2020-06-07 to 2020-06-13)

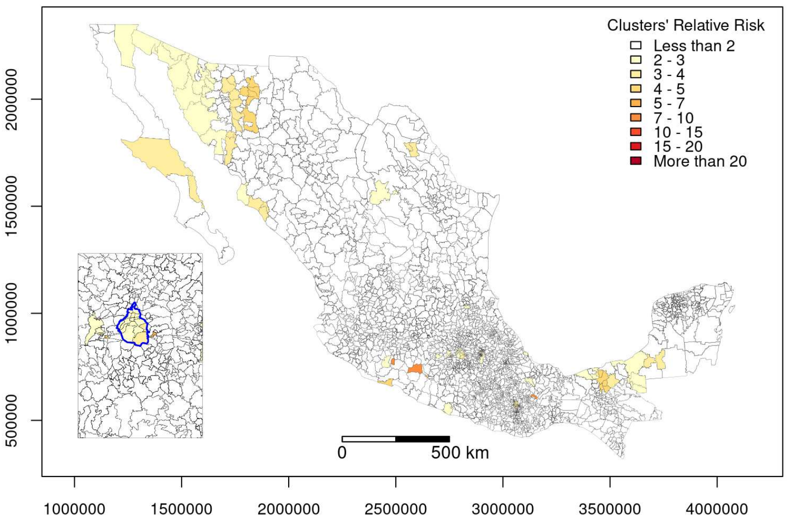

Cases week 26 (2020-06-21 to 2020-06-27)

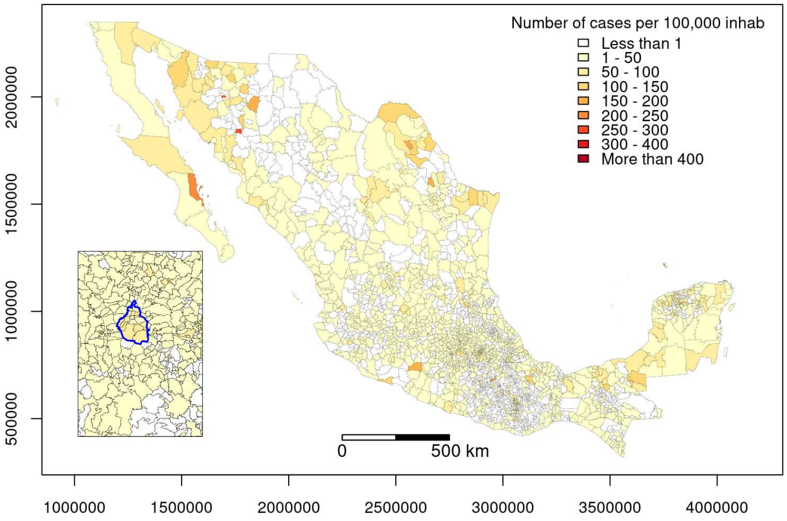

RR week 26 (2020-06-21 to 2020-06-27)

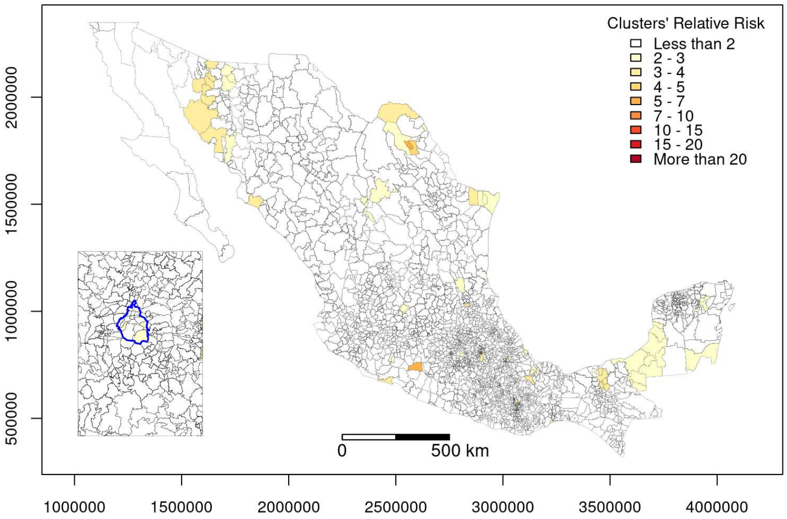

Cases week 28 (2020-07-05 to 2020-07-11)

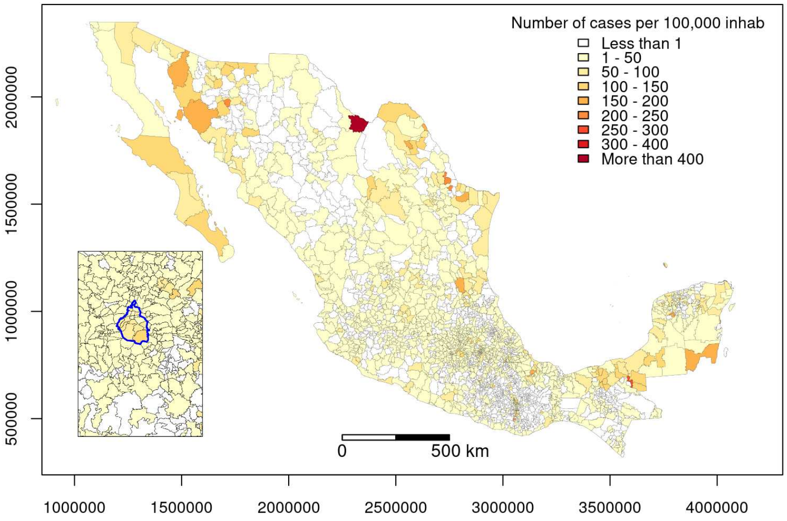

RR week 28 (2020-07-05 to 2020-07-11)

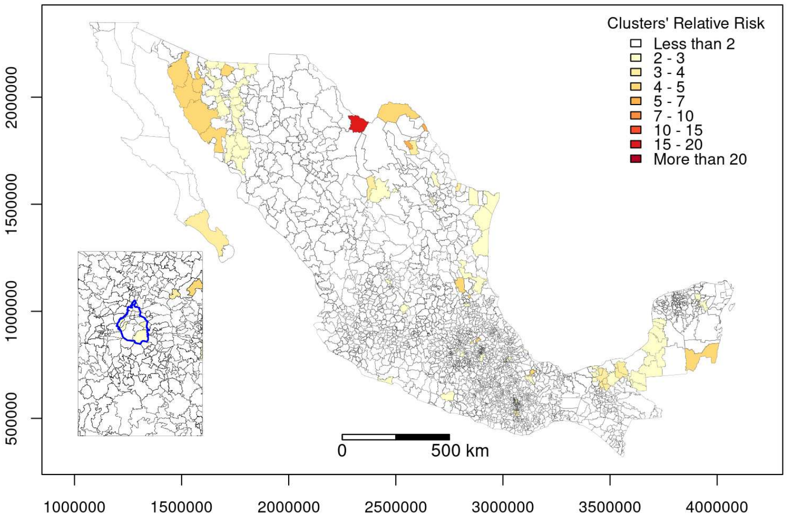

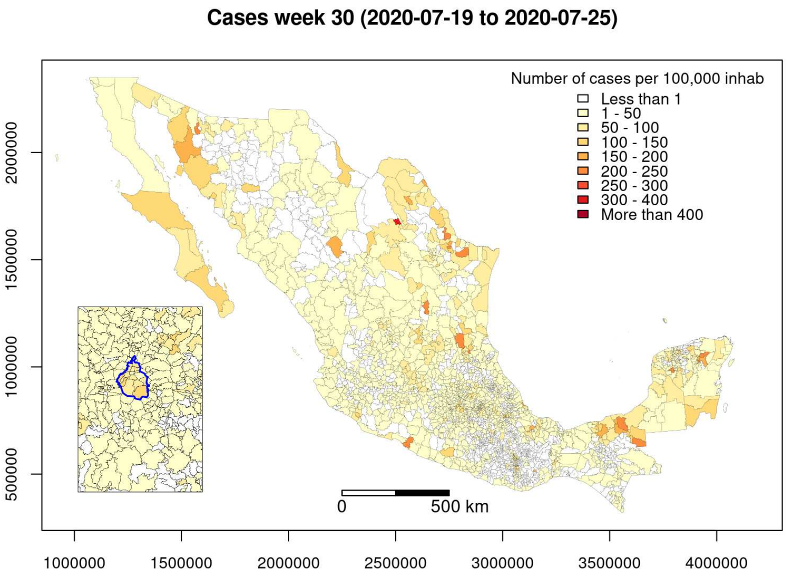
Cases week 30 (2020-07-19 to 2020-07-25)
Number of cases per 100,000 inhab
Less than 1
1 - 50
50 - 100
100 - 150
150 - 200
200 - 250
250 - 300
300 - 400
More than 400
0
500 km
500000
1000000
1500000
2000000
1000000
1500000
2000000
2500000
3000000
3500000
4000000

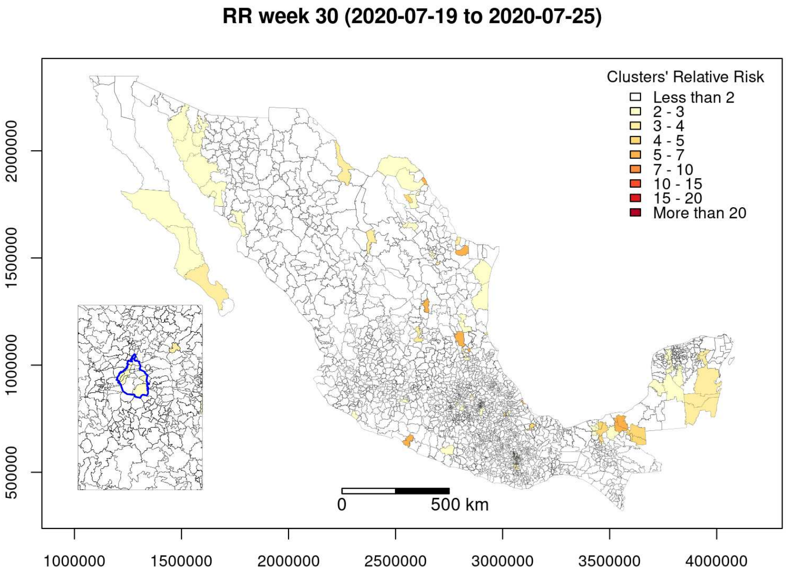
RR week 30 (2020-07-19 to 2020-07-25)
Clusters' Relative Risk
Less than 2
2 - 3
3 - 4
4 - 5
5 - 7
7 - 10
10 - 15
15 - 20
More than 20
0
500 km
500000
1000000
1500000
2000000
1000000
1500000
2000000
2500000
3000000
3500000
4000000

Cases week 32 (2020-08-02 to 2020-08-08)

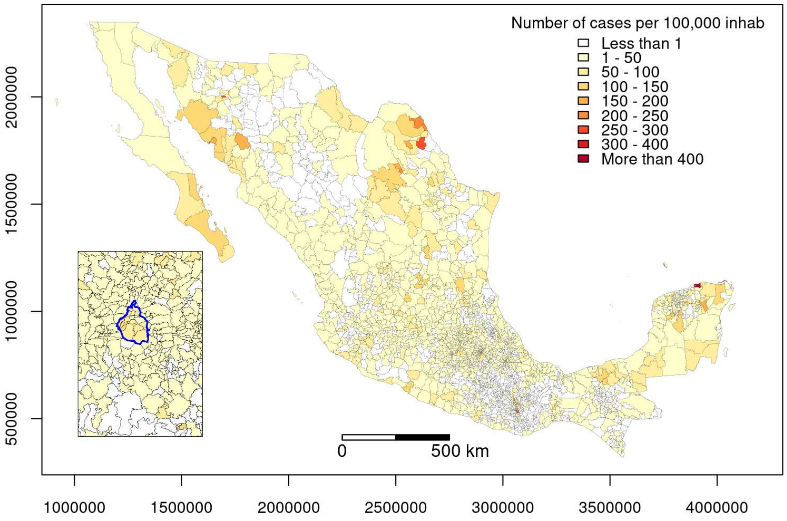

RR week 32 (2020-08-02 to 2020-08-08)

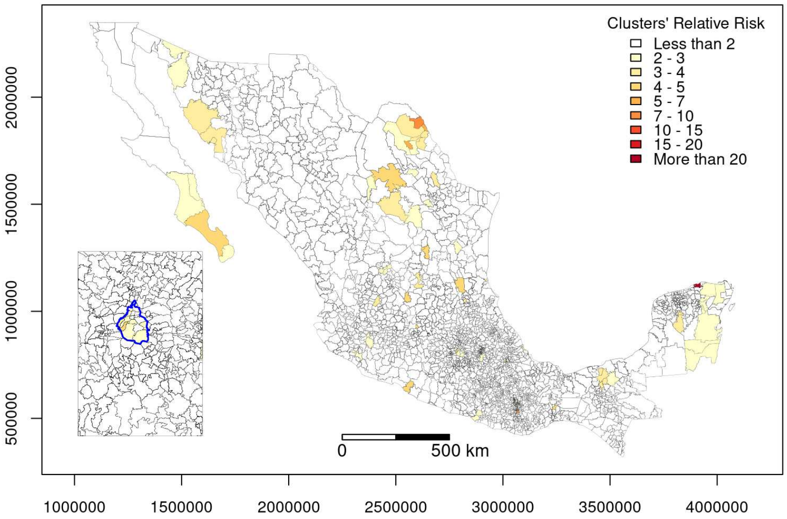

Cases week 34 (2020-08-16 to 2020-08-22)

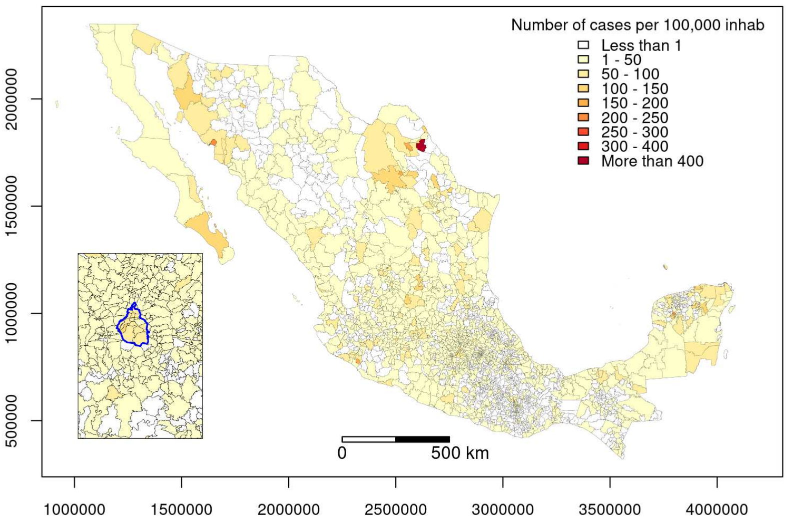

RR week 34 (2020-08-16 to 2020-08-22)

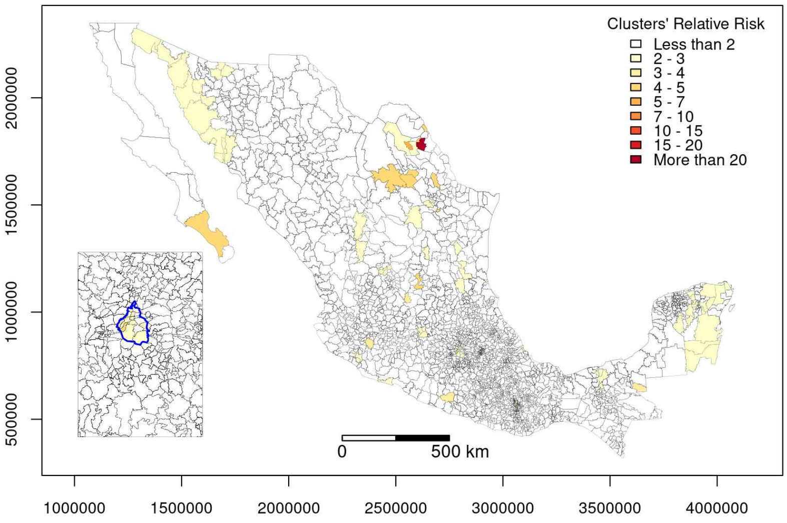

Cases week 36 (2020-08-30 to 2020-09-05)

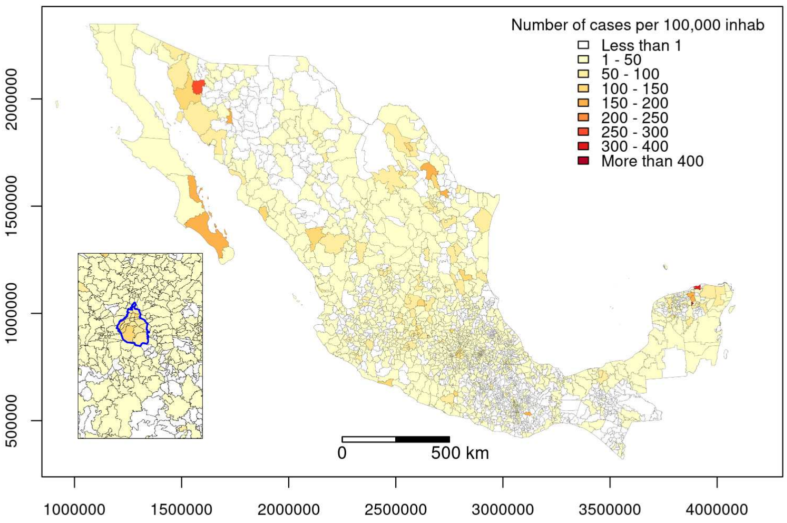

RR week 36 (2020-08-30 to 2020-09-05)

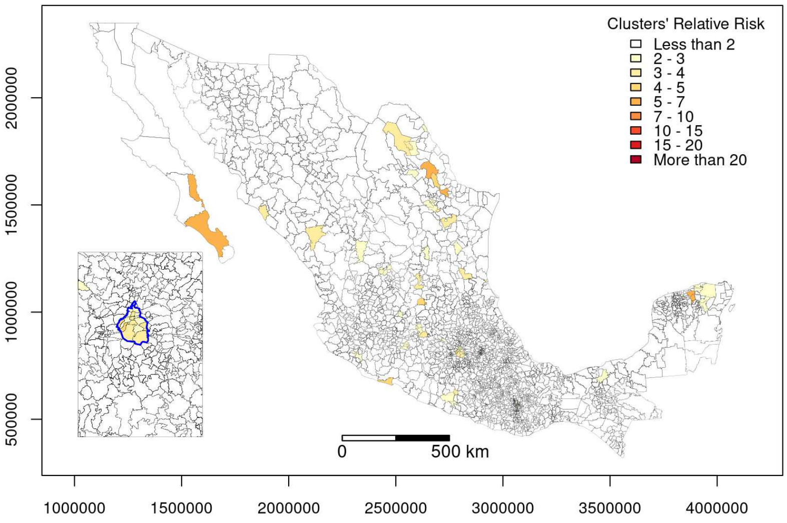

Cases week 38 (2020-09-13 to 2020-09-19)

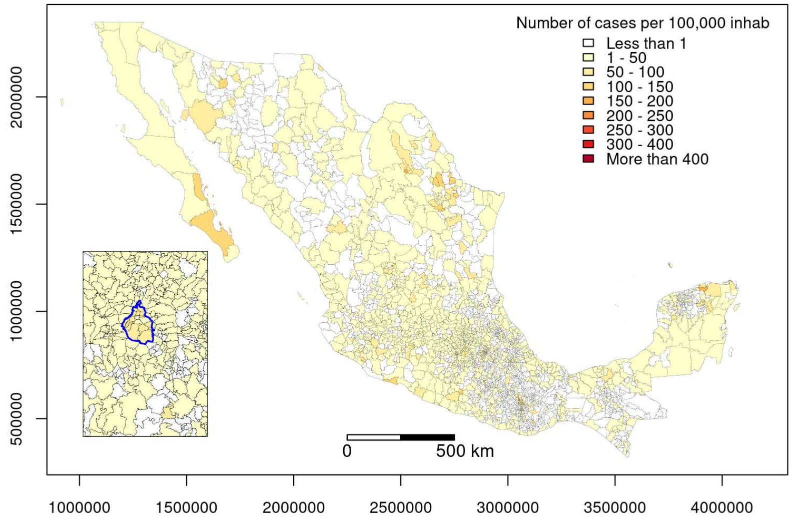

RR week 38 (2020-09-13 to 2020-09-19)

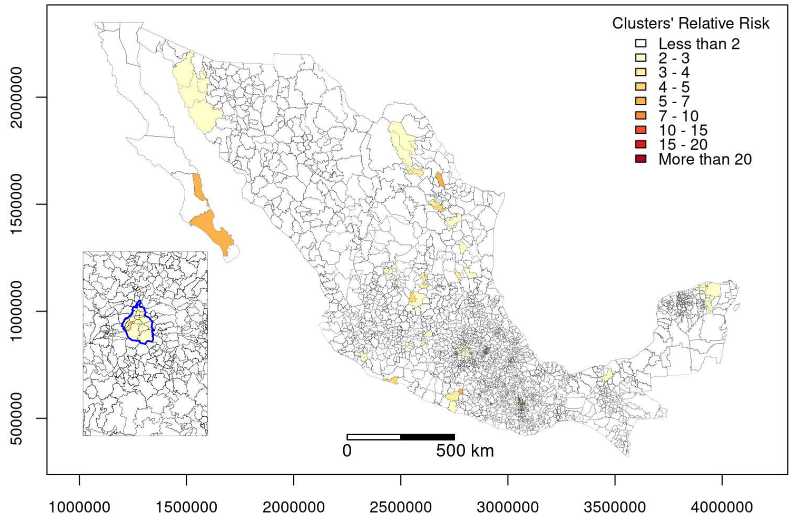

Cases week 40 (2020-09-27 to 2020-10-03)

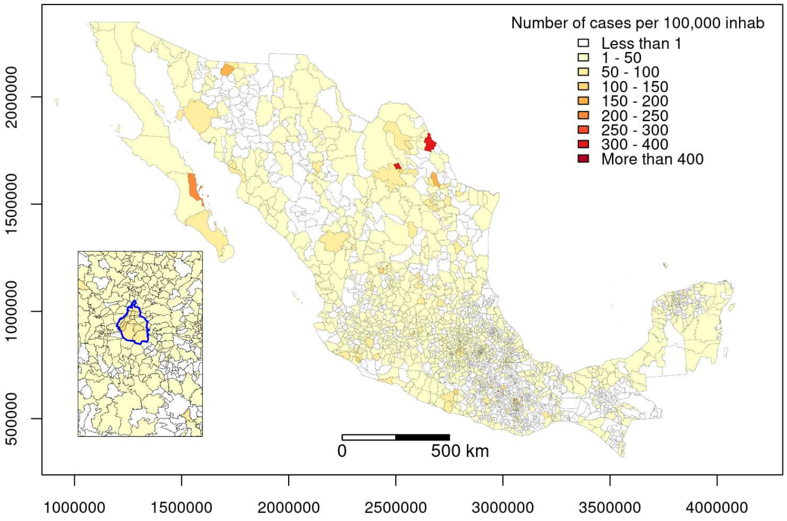

RR week 40 (2020-09-27 to 2020-10-03)

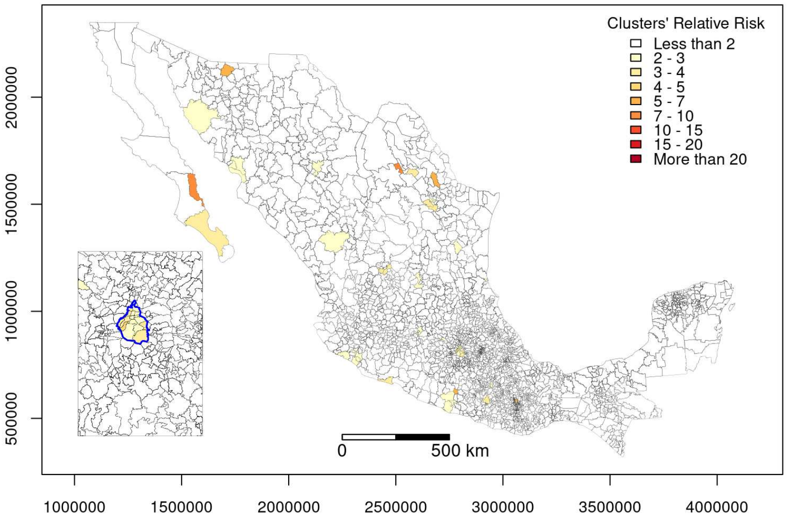

Cases week 42 (2020-10-11 to 2020-10-17)

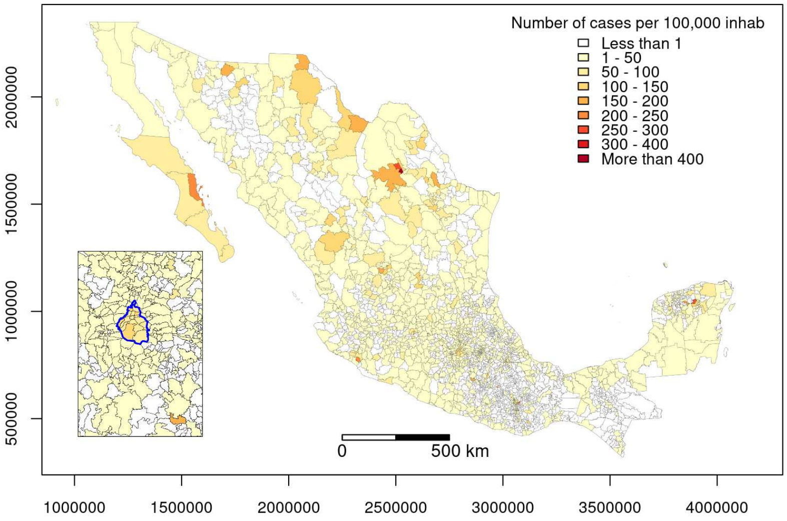

RR week 42 (2020-10-11 to 2020-10-17)

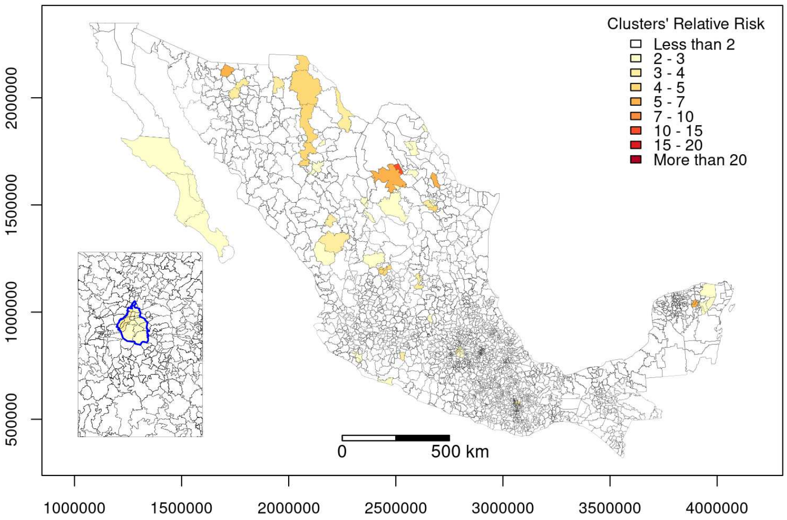

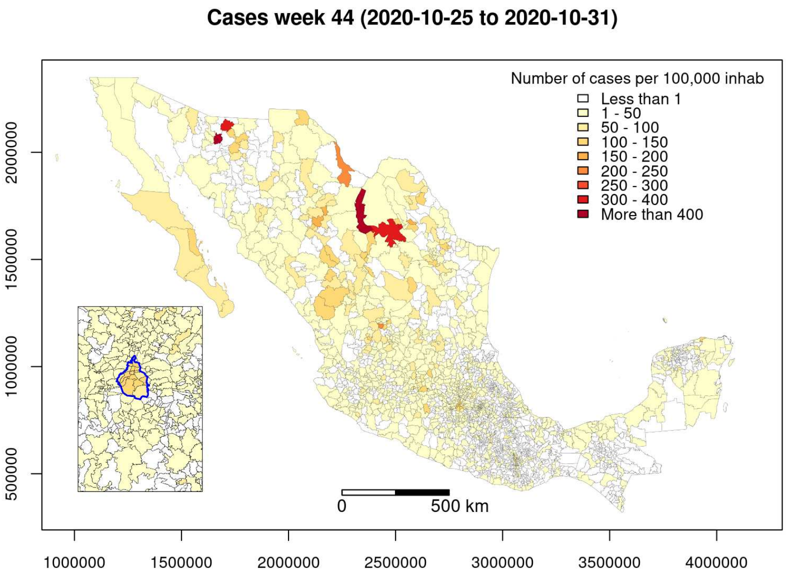
Cases week 44 (2020-10-25 to 2020-10-31)
Number of cases per 100,000 inhab
Less than 1
1 - 50
50 - 100
100 - 150
150 - 200
200 - 250
250 - 300
300 - 400
More than 400
0
500 km
500000
1000000
1500000
2000000
1000000
1500000
2000000
2500000
3000000
3500000
4000000

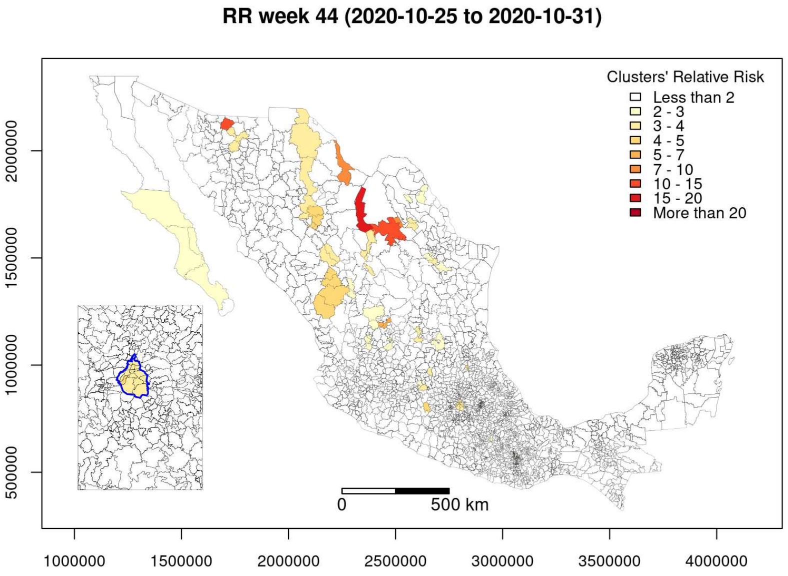
RR week 44 (2020-10-25 to 2020-10-31)
Clusters' Relative Risk
Less than 2
2 - 3
3 - 4
4 - 5
5 - 7
7 - 10
10 - 15
15 - 20
More than 20
0
500 km
500000
1000000
1500000
2000000
1000000
1500000
2000000
2500000
3000000
3500000
4000000

Cases week 46 (2020-11-08 to 2020-11-14)

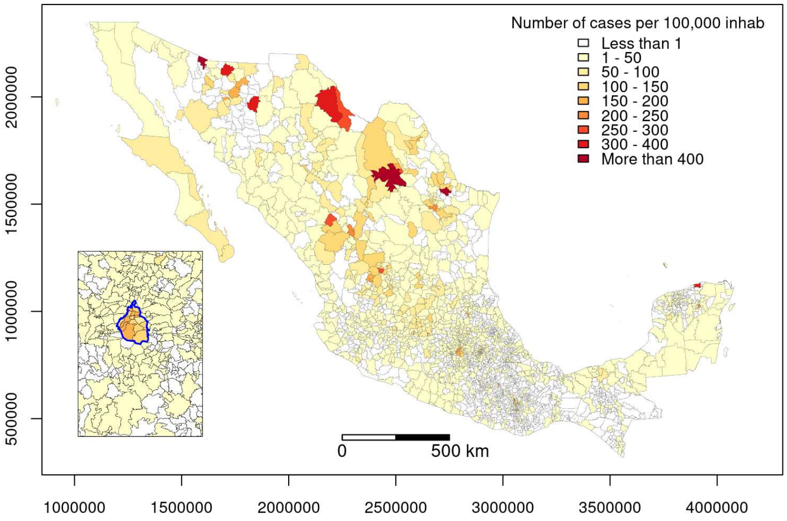

RR week 46 (2020-11-08 to 2020-11-14)

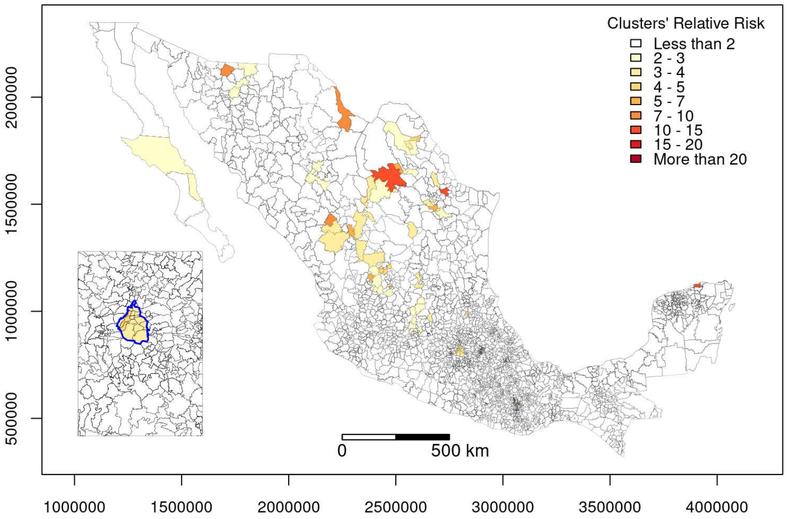

Cases week 48 (2020-11-22 to 2020-11-28)

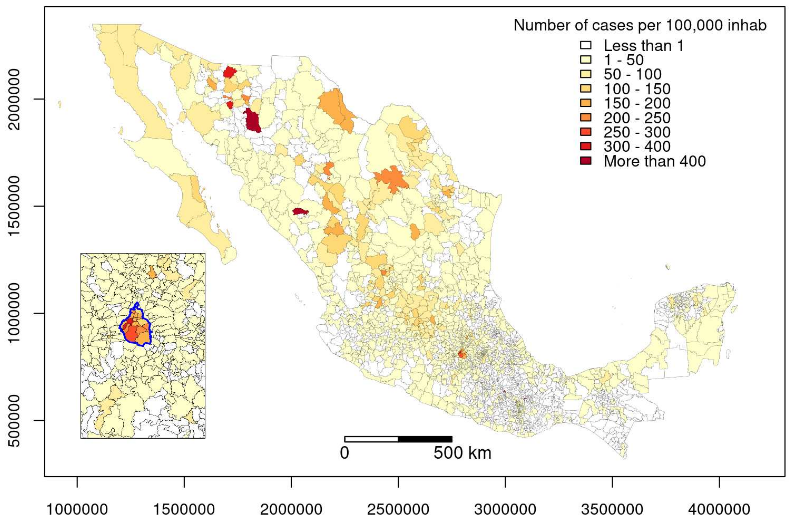

RR week 48 (2020-11-22 to 2020-11-28)

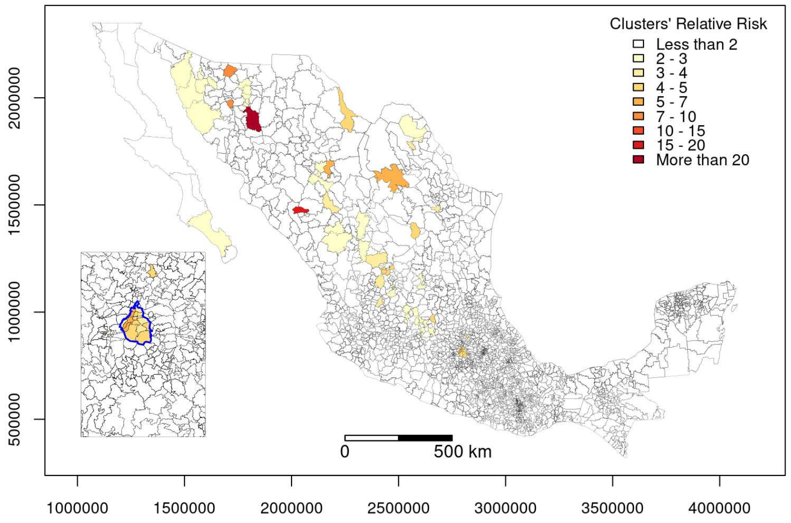

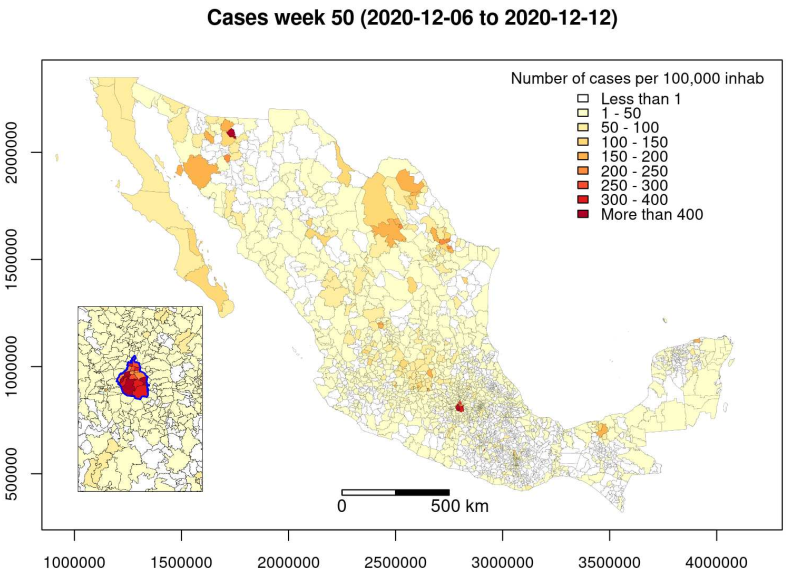
Cases week 50 (2020-12-06 to 2020-12-12)
Number of cases per 100,000 inhab
Less than 1
1 - 50
50 - 100
100 - 150
150 - 200
200 - 250
250 - 300
300 - 400
More than 400
0
500 km
500000
1000000
1500000
2000000
1000000
1500000
2000000
2500000
3000000
3500000
4000000

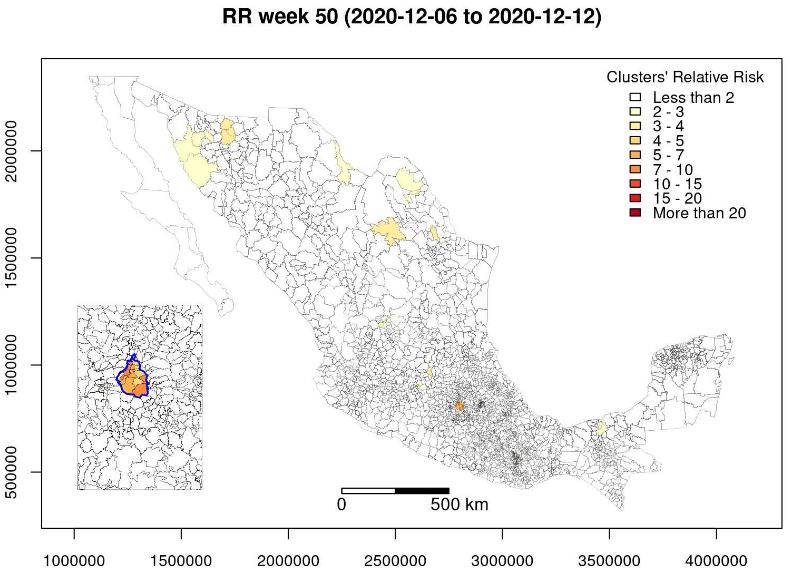
RR week 50 (2020-12-06 to 2020-12-12)
Clusters' Relative Risk
Less than 2
2 - 3
3 - 4
4 - 5
5 - 7
7 - 10
10 - 15
15 - 20
More than 20
0
500 km
500000
1000000
1500000
2000000
1000000
1500000
2000000
2500000
3000000
3500000
4000000

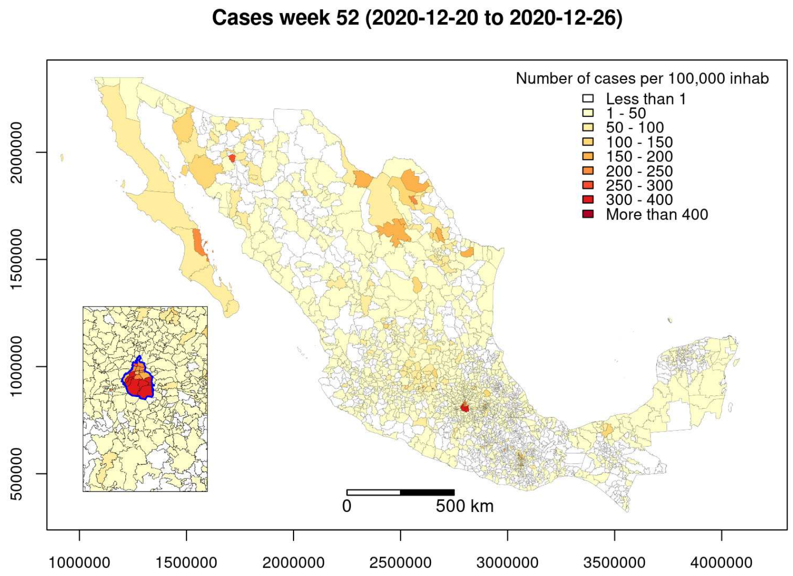

Cases week 52 (2020-12-20 to 2020-12-26)
Number of cases per 100,000 inhab
Less than 1
1 - 50
50 - 100
100 - 150
150 - 200
200 - 250
250 - 300
300 - 400
More than 400
0
500 km
500000
1000000
1500000
2000000
1000000
1500000
2000000
2500000
3000000
3500000
4000000

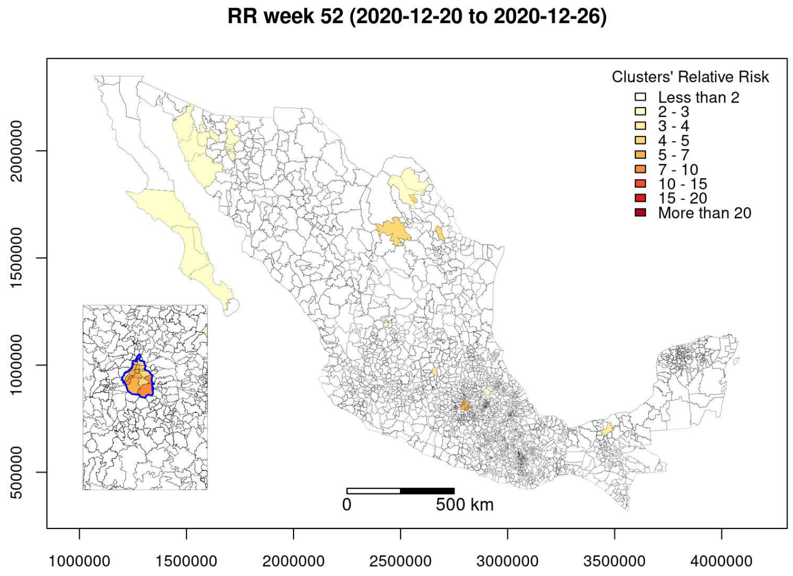

RR week 52 (2020-12-20 to 2020-12-26)
Clusters' Relative Risk
Less than 2
2 - 3
3 - 4
4 - 5
5 - 7
7 - 10
10 - 15
15 - 20
More than 20
0
500 km
500000
1000000
1500000
2000000
1000000
1500000
2000000
2500000
3000000
3500000
4000000

Cases week 54 (2021-01-03 to 2021-01-09)

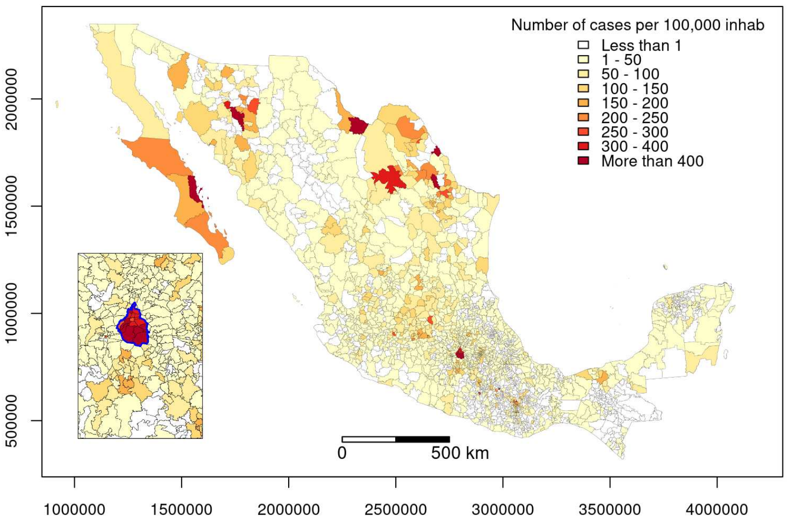

RR week 54 (2021-01-03 to 2021-01-09)

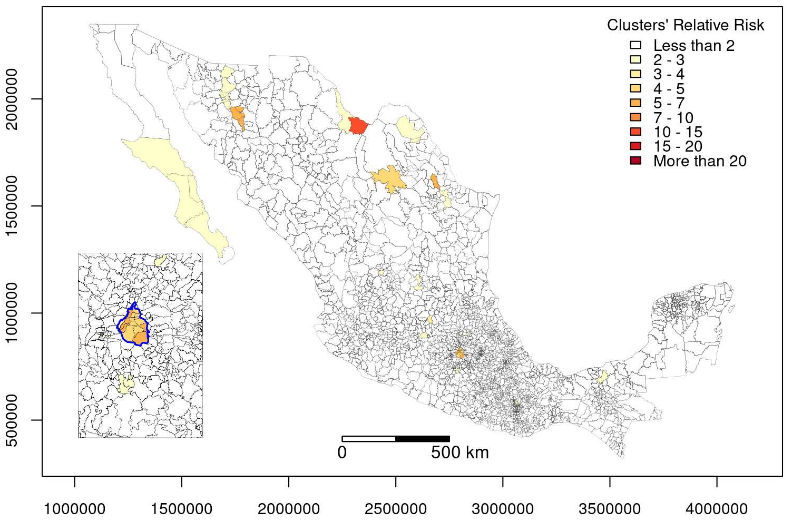

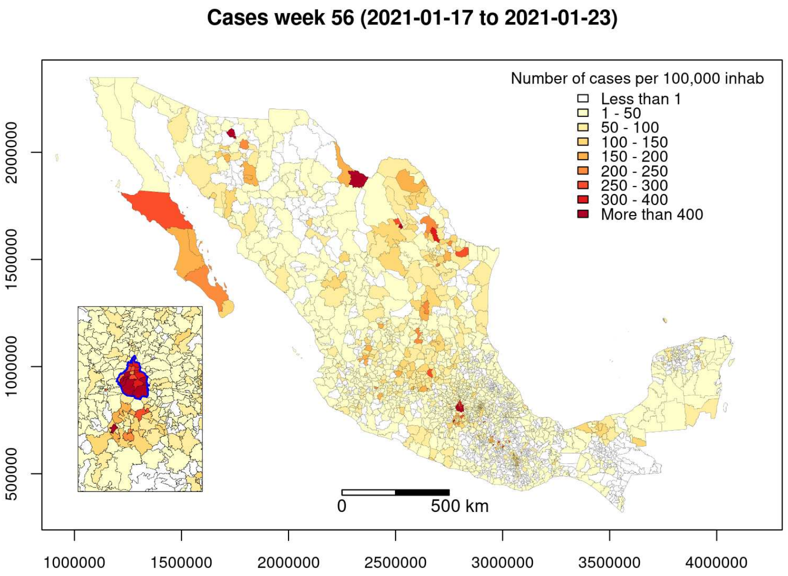

Cases week 56 (2021-01-17 to 2021-01-23)
Number of cases per 100,000 inhab
Less than 1
1 - 50
50 - 100
100 - 150
150 - 200
200 - 250
250 - 300
300 - 400
More than 400
0
500 km
2000000
1500000
1000000
500000
1000000
1500000
2000000
2500000
3000000
3500000
4000000

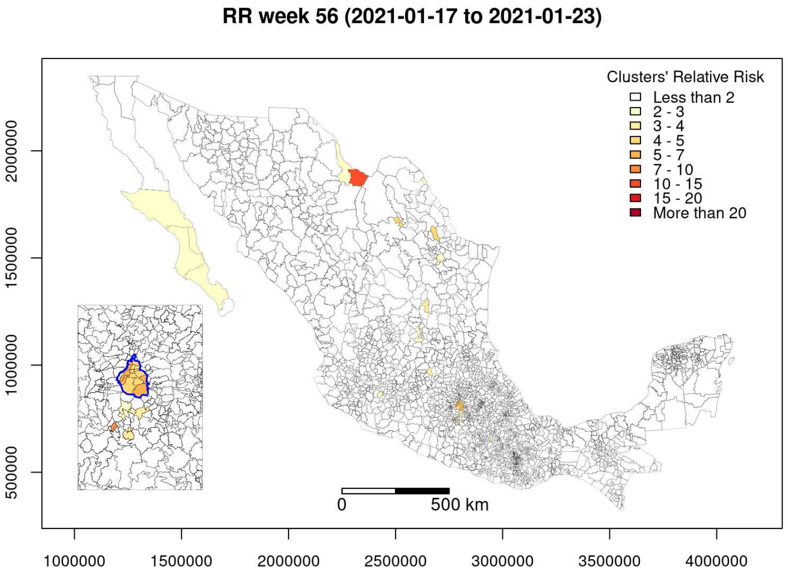

RR week 56 (2021-01-17 to 2021-01-23)
Clusters' Relative Risk
Less than 2
2 - 3
3 - 4
4 - 5
5 - 7
7 - 10
10 - 15
15 - 20
More than 20
0
500 km
2000000
1500000
1000000
500000
1000000
1500000
2000000
2500000
3000000
3500000
4000000

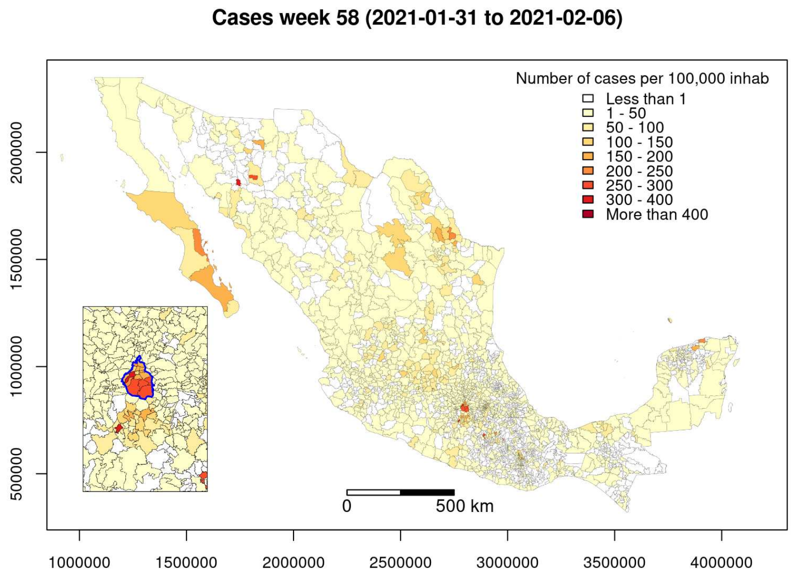
Cases week 58 (2021-01-31 to 2021-02-06)
Number of cases per 100,000 inhab
Less than 1
1 - 50
50 - 100
100 - 150
150 - 200
200 - 250
250 - 300
300 - 400
More than 400
0
500 km
500000
1000000
1500000
2000000
1000000
1500000
2000000
2500000
3000000
3500000
4000000

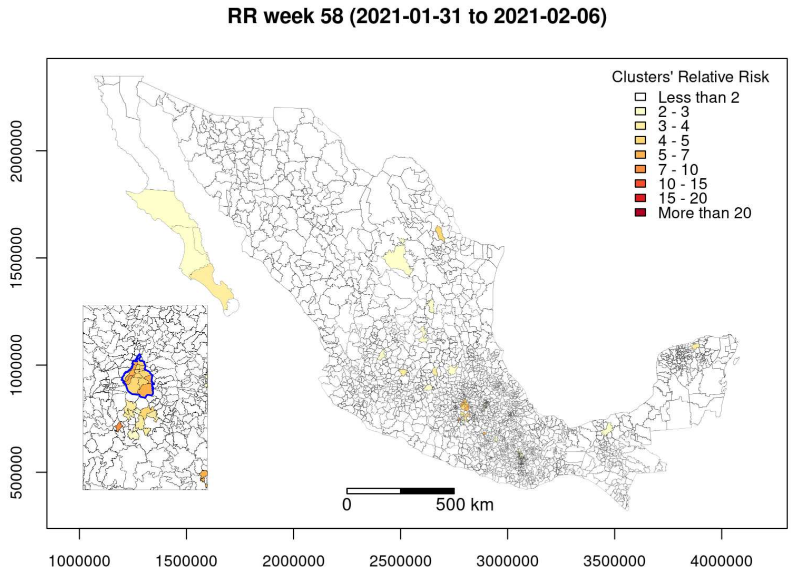
RR week 58 (2021-01-31 to 2021-02-06)
Clusters' Relative Risk
Less than 2
2 - 3
3 - 4
4 - 5
5 - 7
7 - 10
10 - 15
15 - 20
More than 20
0
500 km
500000
1000000
1500000
2000000
1000000
1500000
2000000
2500000
3000000
3500000
4000000

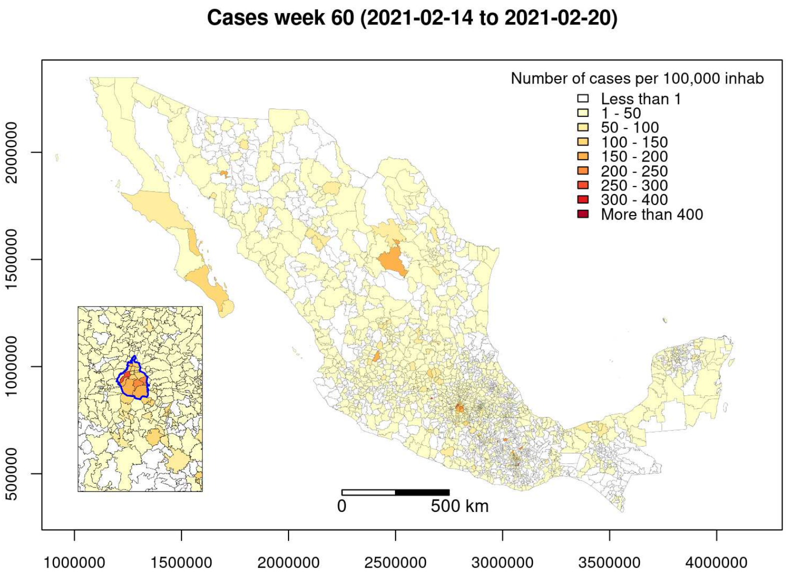
Cases week 60 (2021-02-14 to 2021-02-20)
Number of cases per 100,000 inhab
Less than 1
1 - 50
50 - 100
100 - 150
150 - 200
200 - 250
250 - 300
300 - 400
More than 400
0
500 km
500000
1000000
1500000
2000000
1000000
1500000
2000000
2500000
3000000
3500000
4000000

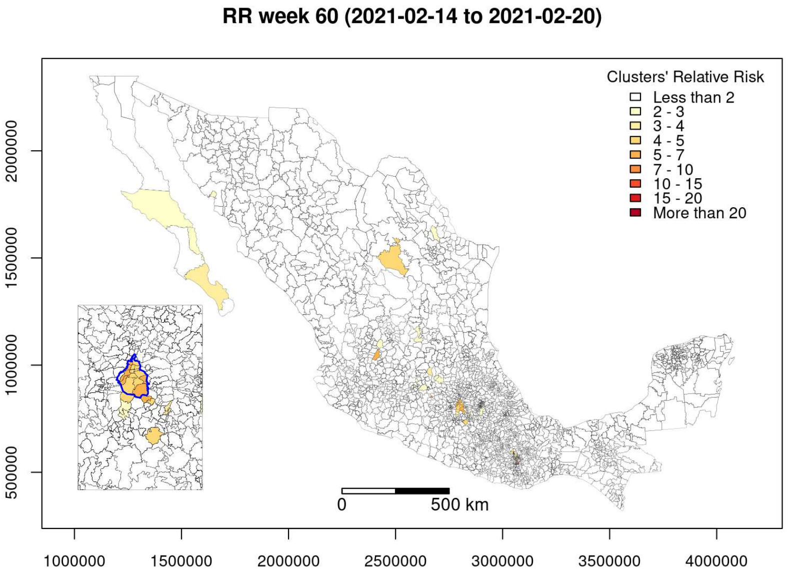
RR week 60 (2021-02-14 to 2021-02-20)
Clusters' Relative Risk
Less than 2
2 - 3
3 - 4
4 - 5
5 - 7
7 - 10
10 - 15
15 - 20
More than 20
0
500 km
500000
1000000
1500000
2000000
1000000
1500000
2000000
2500000
3000000
3500000
4000000

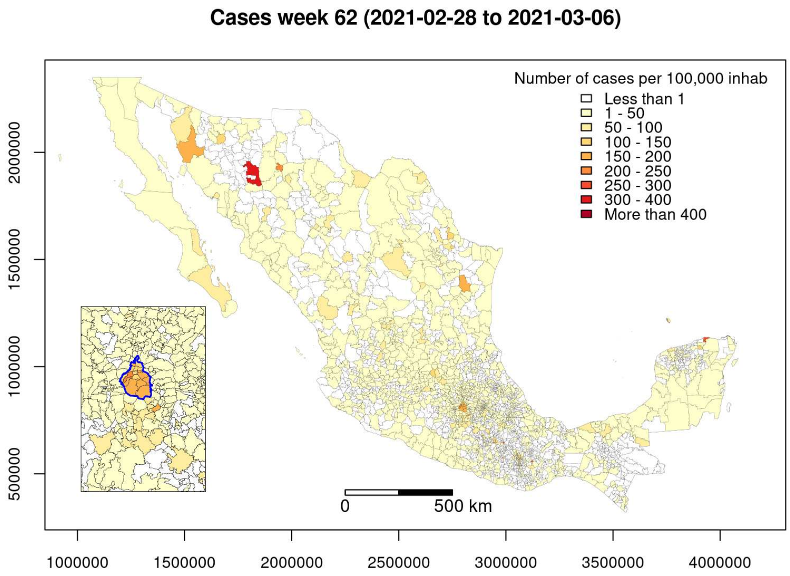
Cases week 62 (2021-02-28 to 2021-03-06)
Number of cases per 100,000 inhab
Less than 1
1 - 50
50 - 100
100 - 150
150 - 200
200 - 250
250 - 300
300 - 400
More than 400
0
500 km
500000
1000000
1500000
2000000
1000000
1500000
2000000
2500000
3000000
3500000
4000000

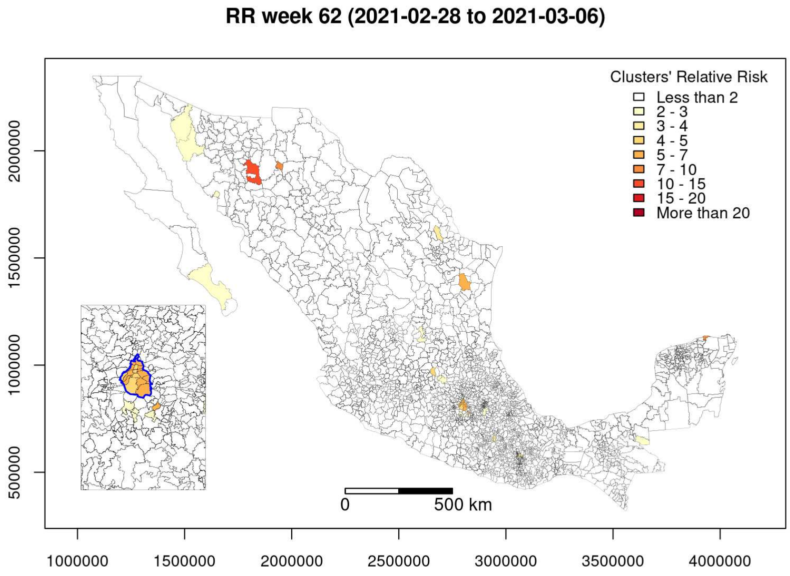
RR week 62 (2021-02-28 to 2021-03-06)
Clusters' Relative Risk
Less than 2
2 - 3
3 - 4
4 - 5
5 - 7
7 - 10
10 - 15
15 - 20
More than 20
0
500 km
500000
1000000
1500000
2000000
1000000
1500000
2000000
2500000
3000000
3500000
4000000

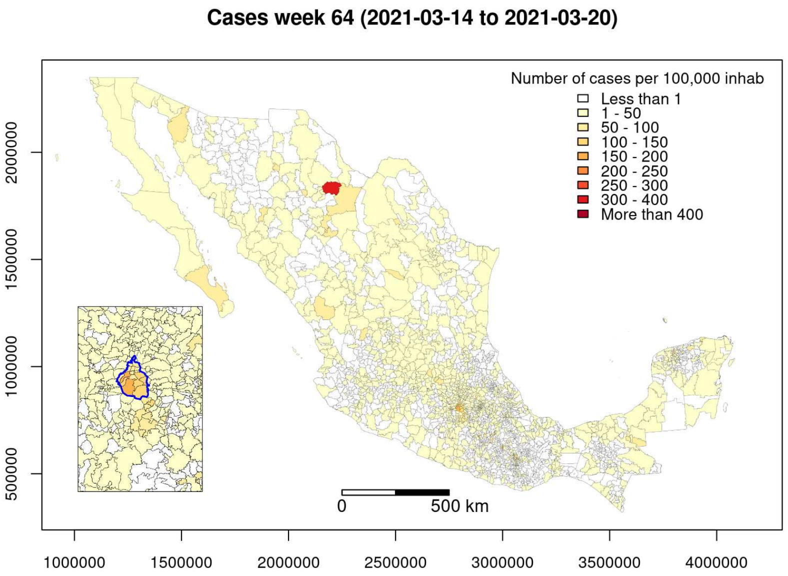
Cases week 64 (2021-03-14 to 2021-03-20)
Number of cases per 100,000 inhab
Less than 1
1 - 50
50 - 100
100 - 150
150 - 200
200 - 250
250 - 300
300 - 400
More than 400
0
500 km
2000000
1500000
1000000
500000
1000000
1500000
2000000
2500000
3000000
3500000
4000000

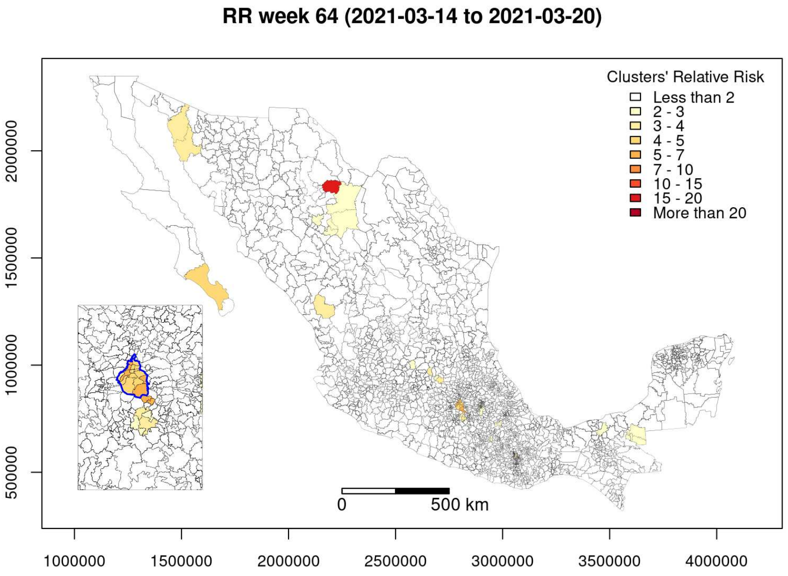
RR week 64 (2021-03-14 to 2021-03-20)
Clusters' Relative Risk
Less than 2
2 - 3
3 - 4
4 - 5
5 - 7
7 - 10
10 - 15
15 - 20
More than 20
0
500 km
2000000
1500000
1000000
500000
1000000
1500000
2000000
2500000
3000000
3500000
4000000

Cases week 66 (2021-03-28 to 2021-04-03)

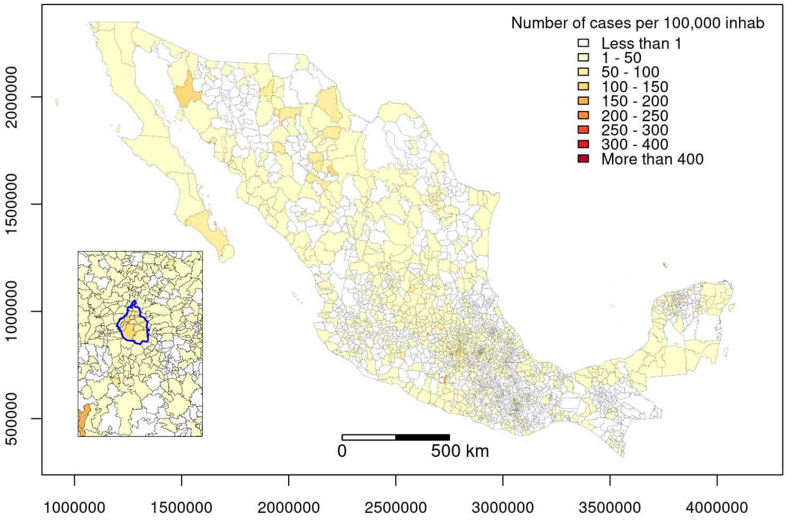

RR week 66 (2021-03-28 to 2021-04-03)

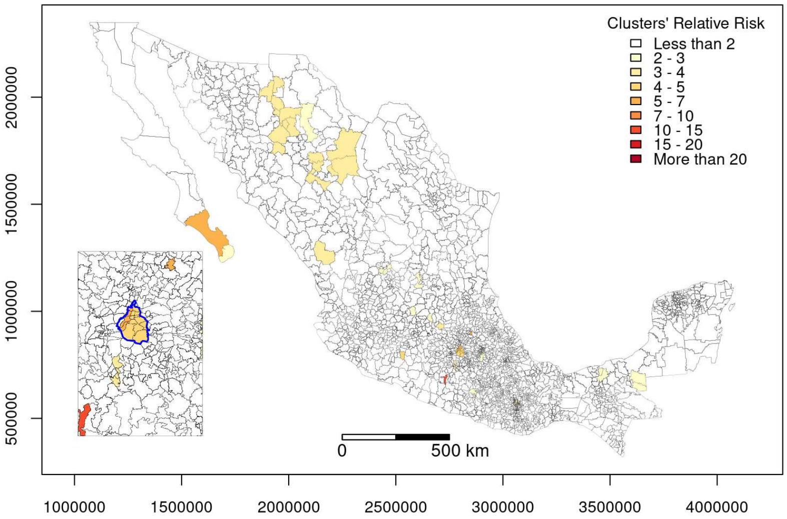

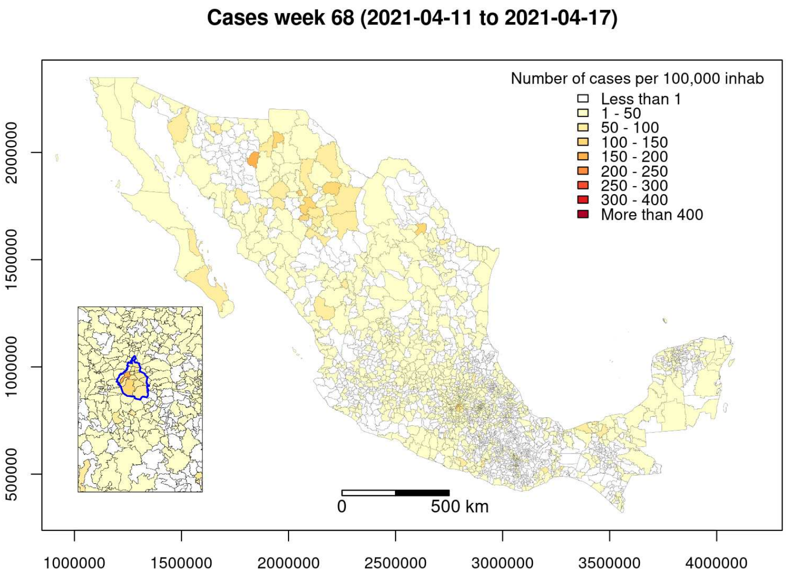
Cases week 68 (2021-04-11 to 2021-04-17)
Number of cases per 100,000 inhab
Less than 1
1 - 50
50 - 100
100 - 150
150 - 200
200 - 250
250 - 300
300 - 400
More than 400
0
500 km
2000000
1500000
1000000
500000
1000000
1500000
2000000
2500000
3000000
3500000
4000000

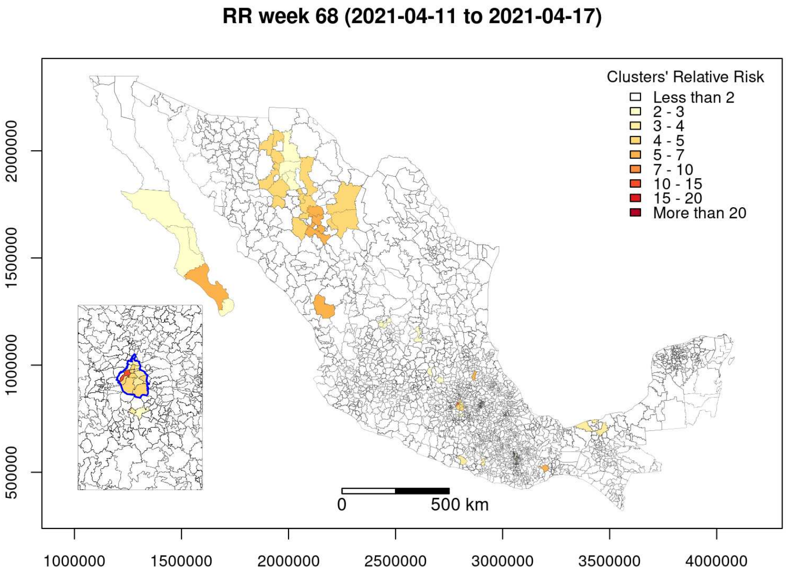
RR week 68 (2021-04-11 to 2021-04-17)
Clusters' Relative Risk
Less than 2
2 - 3
3 - 4
4 - 5
5 - 7
7 - 10
10 - 15
15 - 20
More than 20
0
500 km
2000000
1500000
1000000
500000
1000000
1500000
2000000
2500000
3000000
3500000
4000000

Cases week 70 (2021-04-25 to 2021-05-01)

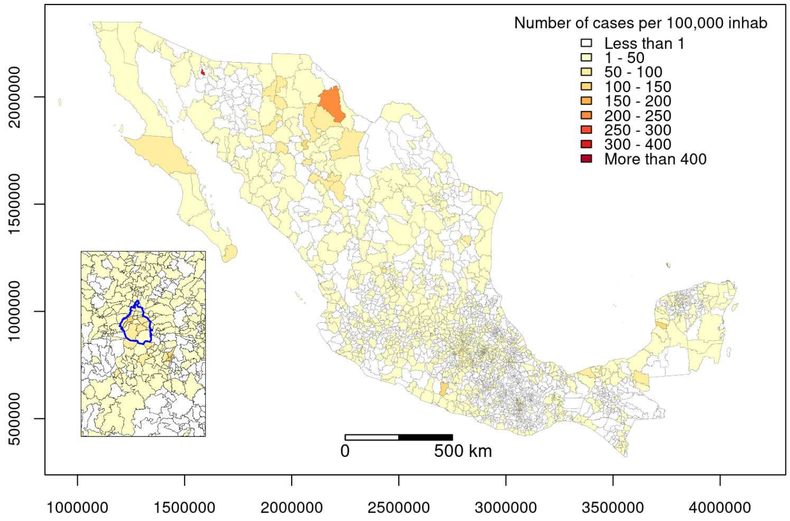

RR week 70 (2021-04-25 to 2021-05-01)

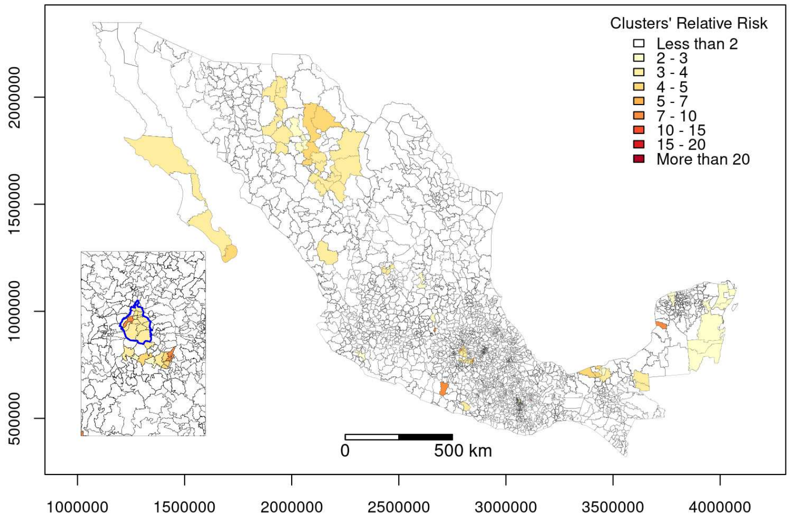

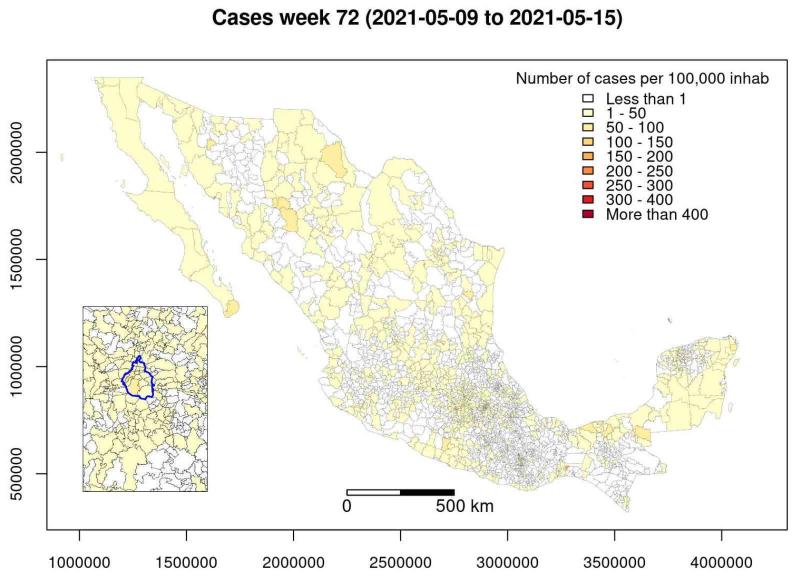
Cases week 72 (2021-05-09 to 2021-05-15)
Number of cases per 100,000 inhab
Less than 1
1 - 50
50 - 100
100 - 150
150 - 200
200 - 250
250 - 300
300 - 400
More than 400
0
500 km
2000000
1500000
1000000
500000
1000000
1500000
2000000
2500000
3000000
3500000
4000000

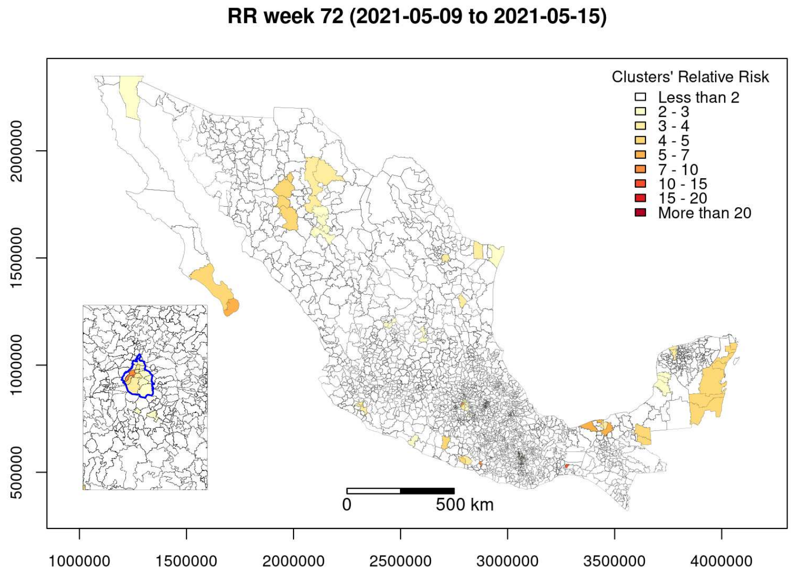
RR week 72 (2021-05-09 to 2021-05-15)
Clusters' Relative Risk
Less than 2
2 - 3
3 - 4
4 - 5
5 - 7
7 - 10
10 - 15
15 - 20
More than 20
0
500 km
2000000
1500000
1000000
500000
1000000
1500000
2000000
2500000
3000000
3500000
4000000

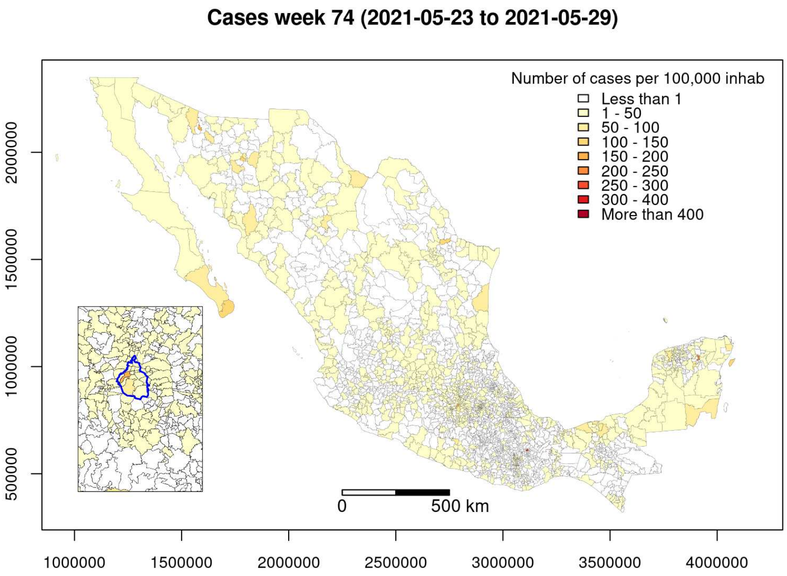
Cases week 74 (2021-05-23 to 2021-05-29)
Number of cases per 100,000 inhab
Less than 1
1 - 50
50 - 100
100 - 150
150 - 200
200 - 250
250 - 300
300 - 400
More than 400
0
500 km
500000
1000000
1500000
2000000
1000000
1500000
2000000
2500000
3000000
3500000
4000000

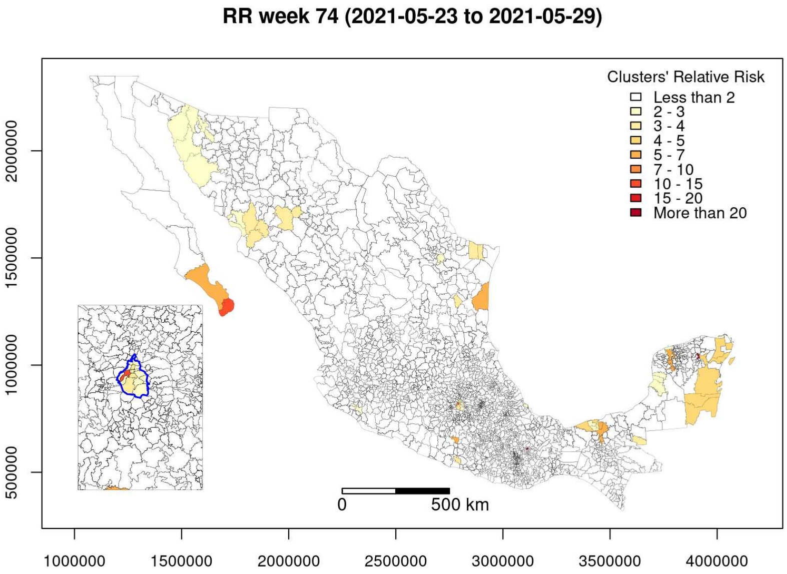
RR week 74 (2021-05-23 to 2021-05-29)
Clusters' Relative Risk
Less than 2
2 - 3
3 - 4
4 - 5
5 - 7
7 - 10
10 - 15
15 - 20
More than 20
0
500 km
500000
1000000
1500000
2000000
1000000
1500000
2000000
2500000
3000000
3500000
4000000

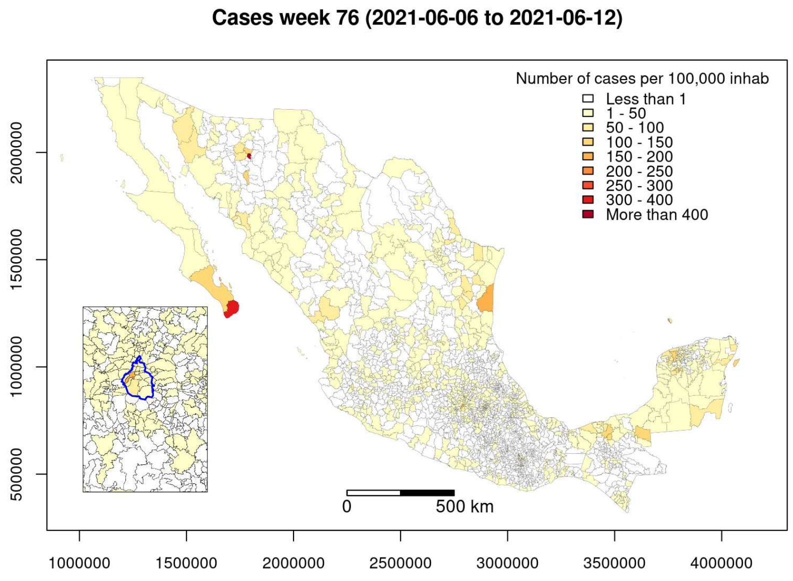
Cases week 76 (2021-06-06 to 2021-06-12)
Number of cases per 100,000 inhab
Less than 1
1 - 50
50 - 100
100 - 150
150 - 200
200 - 250
250 - 300
300 - 400
More than 400
0
500 km
500000
1000000
1500000
2000000
1000000
1500000
2000000
2500000
3000000
3500000
4000000

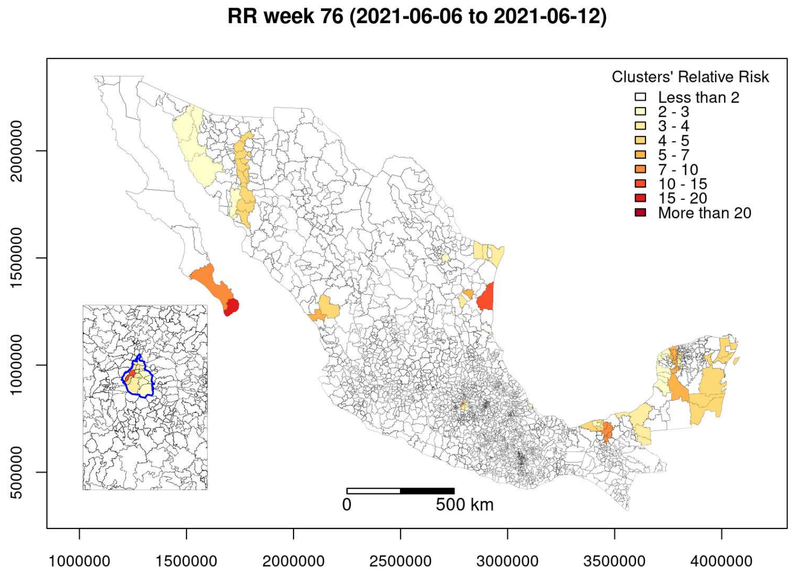
RR week 76 (2021-06-06 to 2021-06-12)
Clusters' Relative Risk
Less than 2
2 - 3
3 - 4
4 - 5
5 - 7
7 - 10
10 - 15
15 - 20
More than 20
0
500 km
500000
1000000
1500000
2000000
1000000
1500000
2000000
2500000
3000000
3500000
4000000

Cases week 78 (2021-06-20 to 2021-06-26)

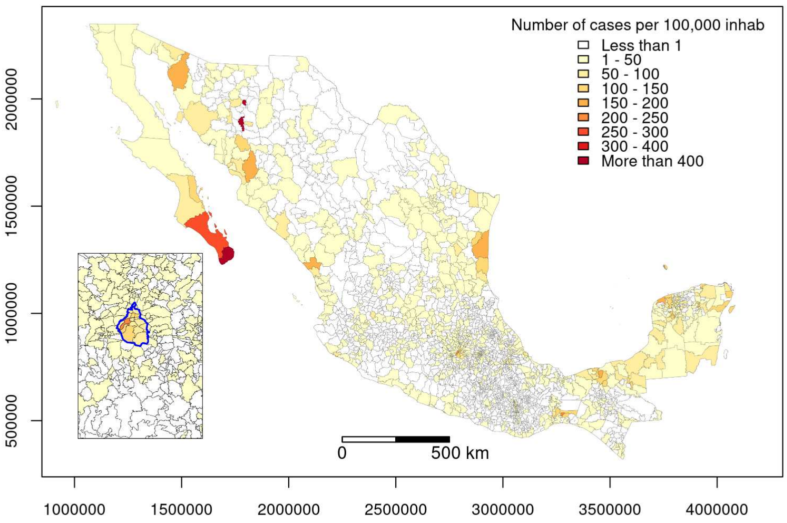

RR week 78 (2021-06-20 to 2021-06-26)

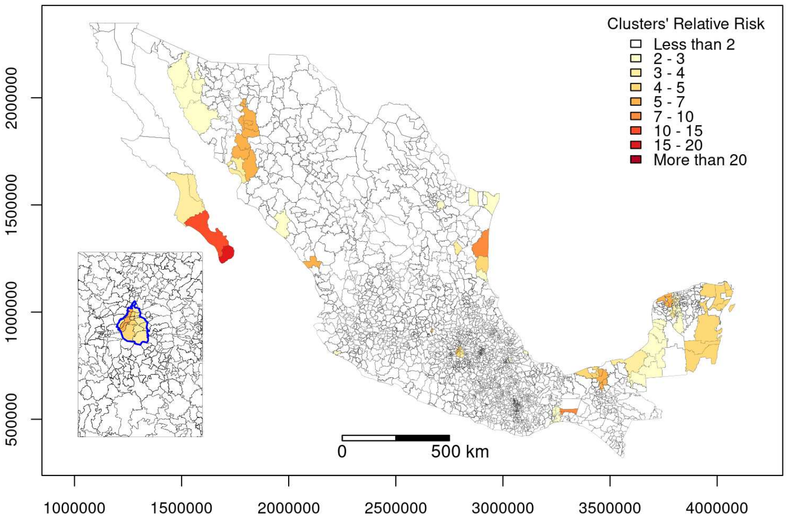

Cases week 80 (2021-07-04 to 2021-07-10)

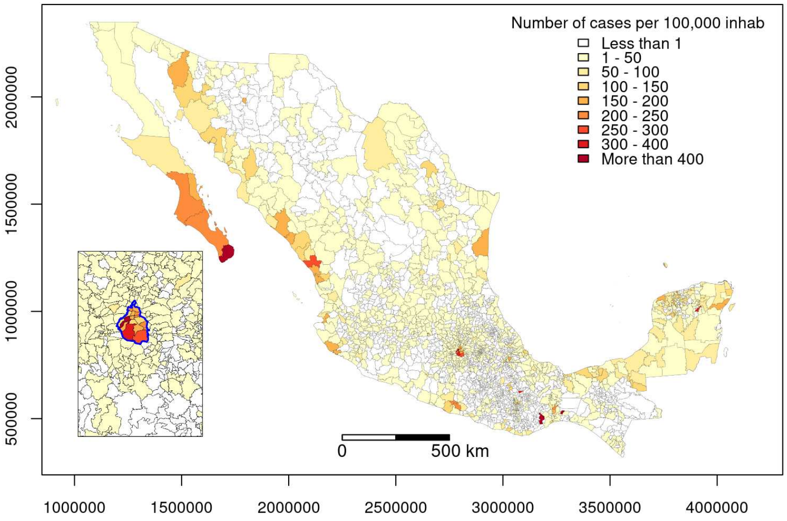

RR week 80 (2021-07-04 to 2021-07-10)

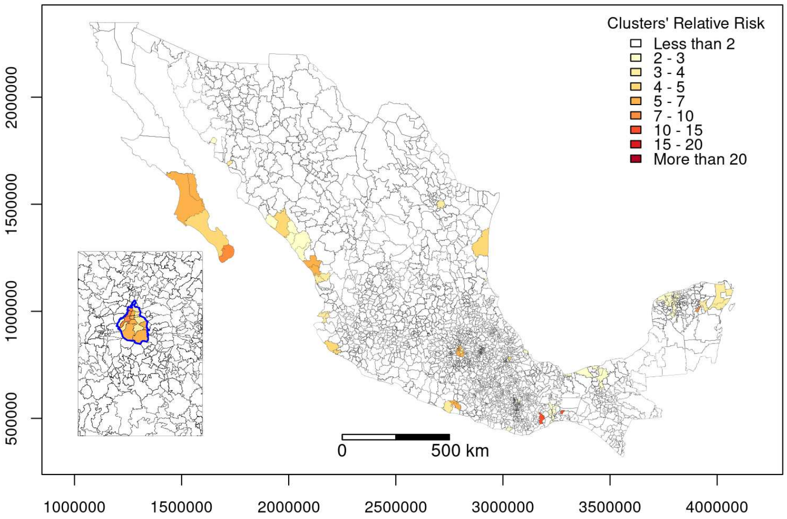

Cases week 82 (2021-07-18 to 2021-07-24)

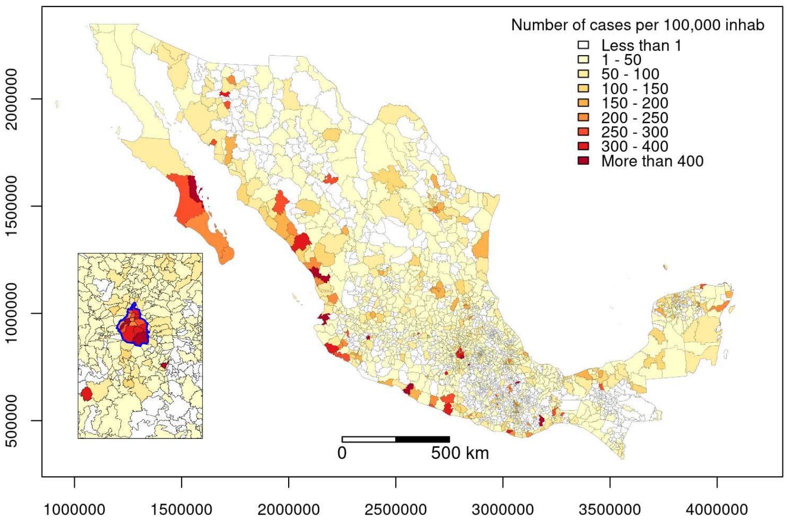

RR week 82 (2021-07-18 to 2021-07-24)

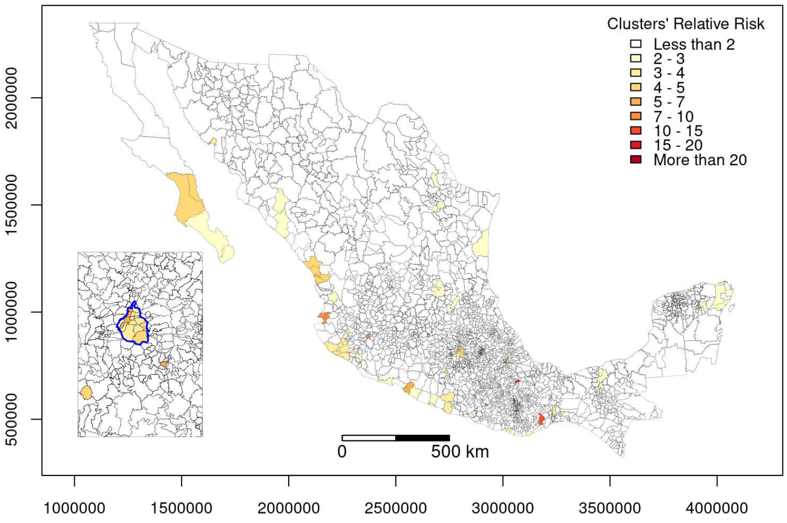

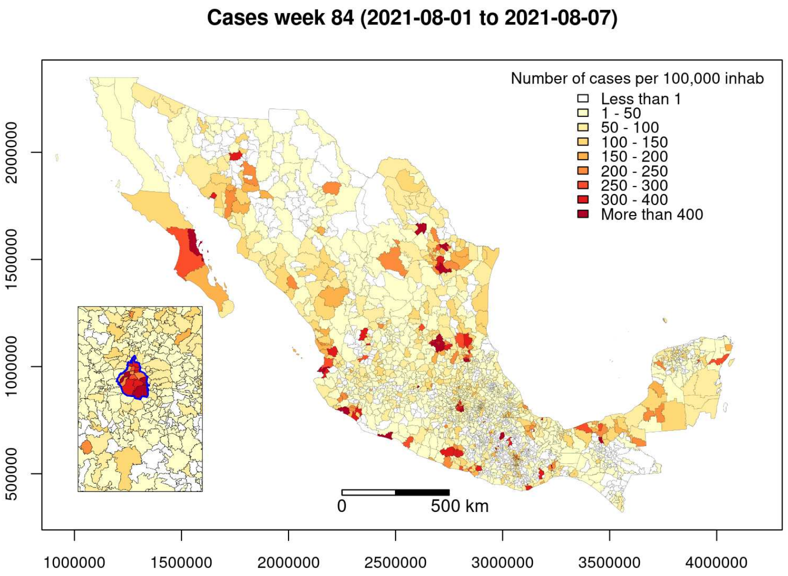
Cases week 84 (2021-08-01 to 2021-08-07)
Number of cases per 100,000 inhab
Less than 1
1 - 50
50 - 100
100 - 150
150 - 200
200 - 250
250 - 300
300 - 400
More than 400
0
500 km
500000
1000000
1500000
2000000
1000000
1500000
2000000
2500000
3000000
3500000
4000000

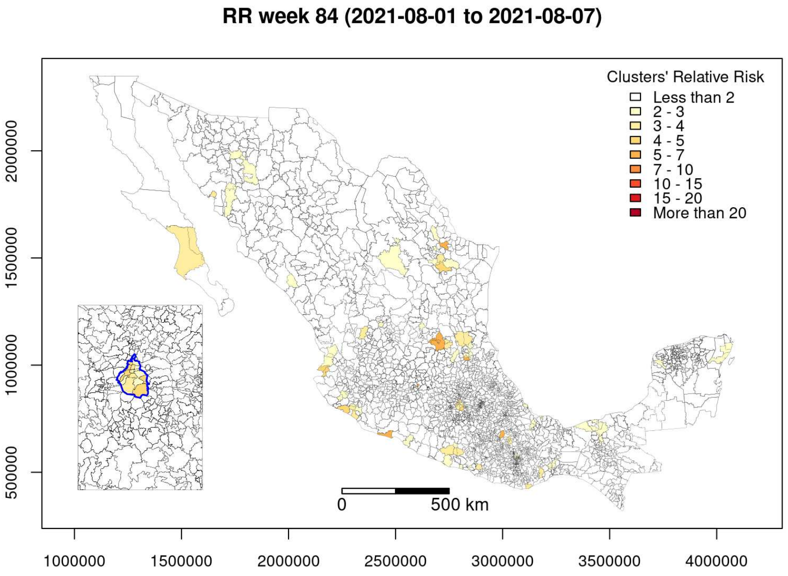
RR week 84 (2021-08-01 to 2021-08-07)
Clusters' Relative Risk
Less than 2
2 - 3
3 - 4
4 - 5
5 - 7
7 - 10
10 - 15
15 - 20
More than 20
0
500 km
500000
1000000
1500000
2000000
1000000
1500000
2000000
2500000
3000000
3500000
4000000

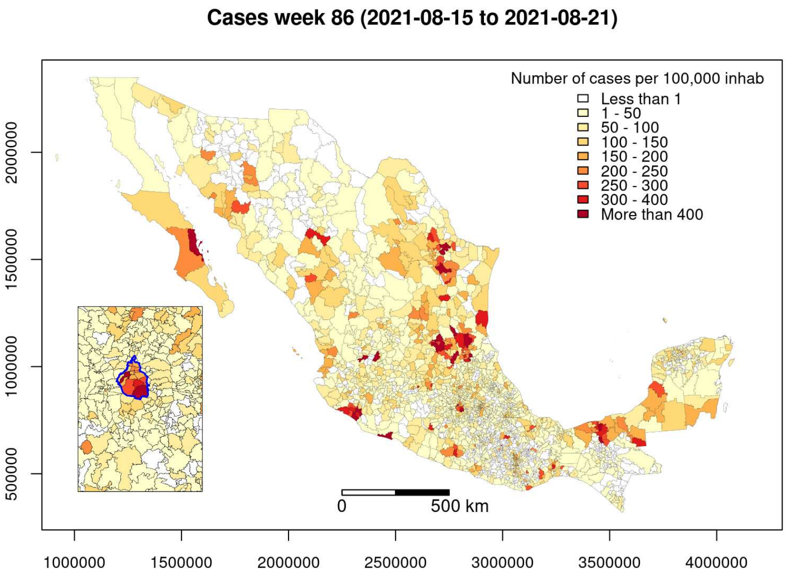
Cases week 86 (2021-08-15 to 2021-08-21)
Number of cases per 100,000 inhab
Less than 1
1 - 50
50 - 100
100 - 150
150 - 200
200 - 250
250 - 300
300 - 400
More than 400
0
500 km
500000
1000000
1500000
2000000
1000000
1500000
2000000
2500000
3000000
3500000
4000000

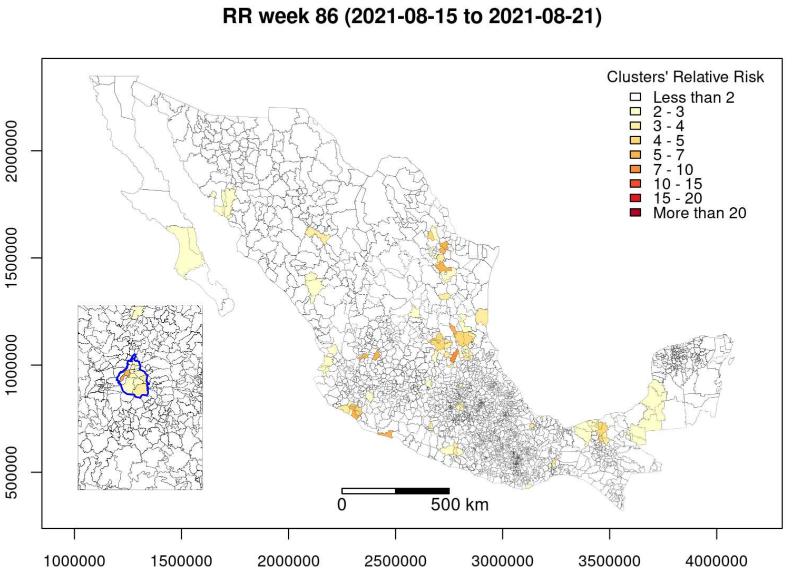
RR week 86 (2021-08-15 to 2021-08-21)
Clusters' Relative Risk
Less than 2
2 - 3
3 - 4
4 - 5
5 - 7
7 - 10
10 - 15
15 - 20
More than 20
0
500 km
500000
1000000
1500000
2000000
1000000
1500000
2000000
2500000
3000000
3500000
4000000

Cases week 88 (2021-08-29 to 2021-09-04)

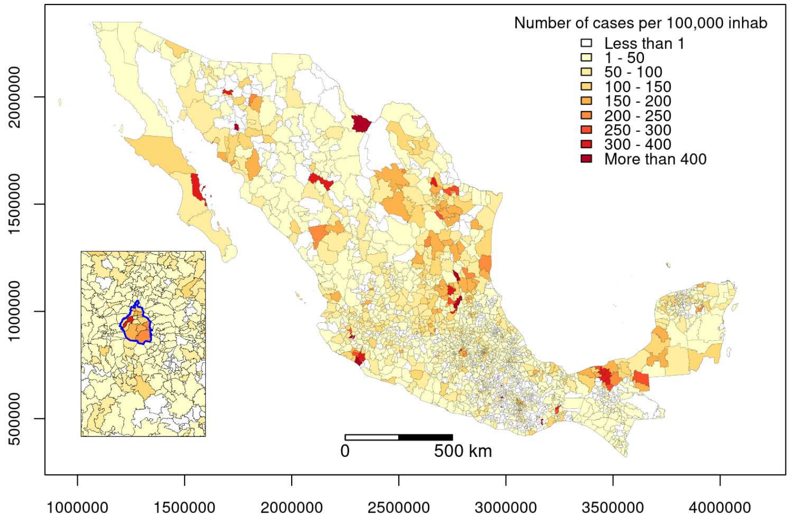

RR week 88 (2021-08-29 to 2021-09-04)

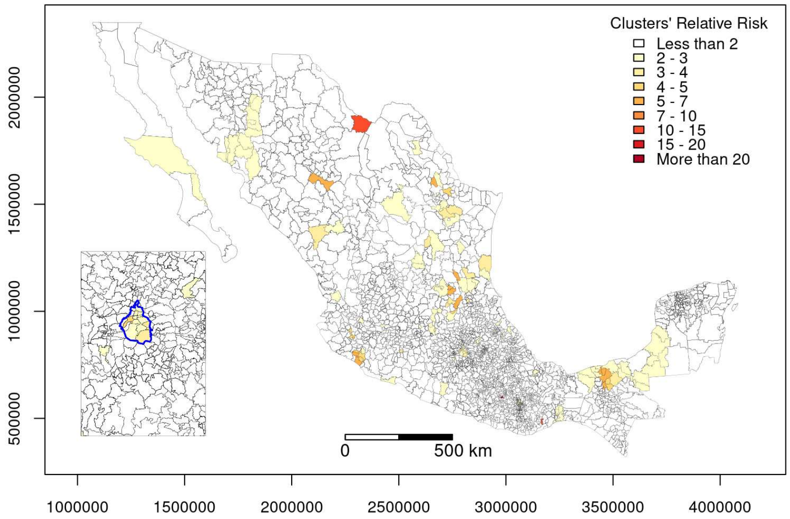

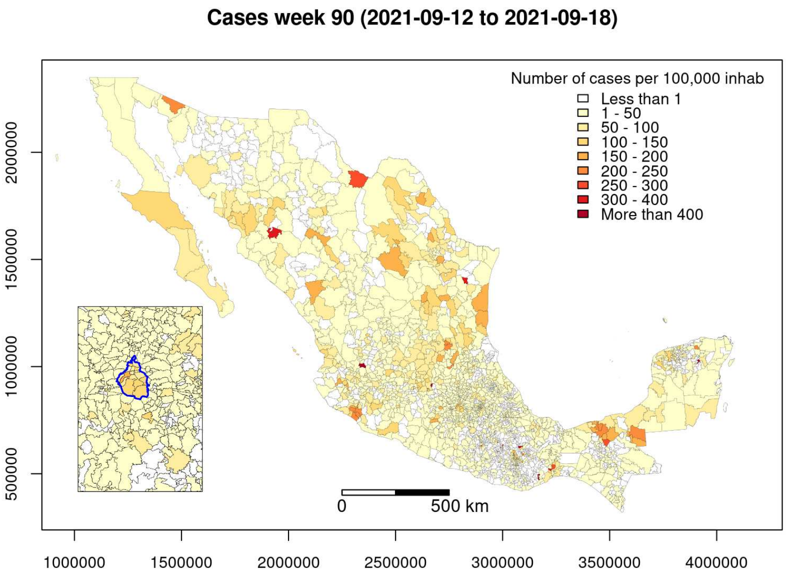
Cases week 90 (2021-09-12 to 2021-09-18)
Number of cases per 100,000 inhab
Less than 1
1 - 50
50 - 100
100 - 150
150 - 200
200 - 250
250 - 300
300 - 400
More than 400
0
500 km
500000
1000000
1500000
2000000
1000000
1500000
2000000
2500000
3000000
3500000
4000000

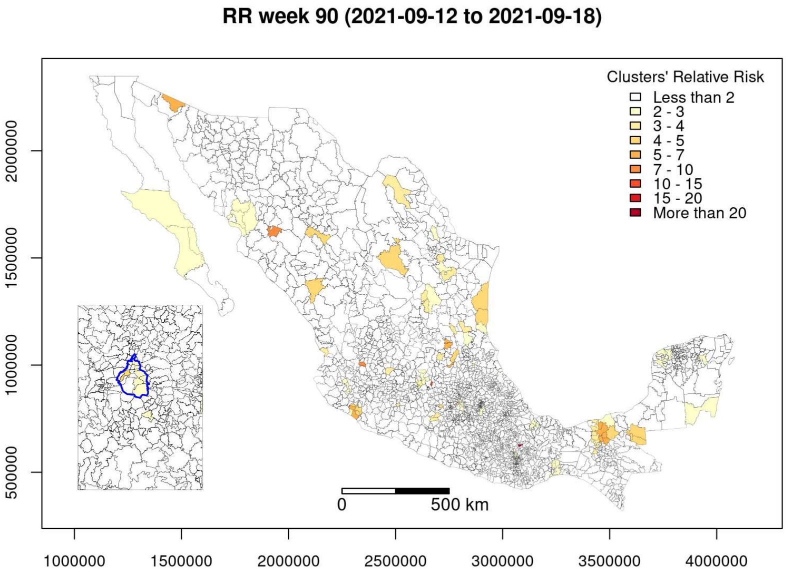
RR week 90 (2021-09-12 to 2021-09-18)
Clusters' Relative Risk
Less than 2
2 - 3
3 - 4
4 - 5
5 - 7
7 - 10
10 - 15
15 - 20
More than 20
0
500 km
500000
1000000
1500000
2000000
1000000
1500000
2000000
2500000
3000000
3500000
4000000

Cases week 92 (2021-09-26 to 2021-10-02)

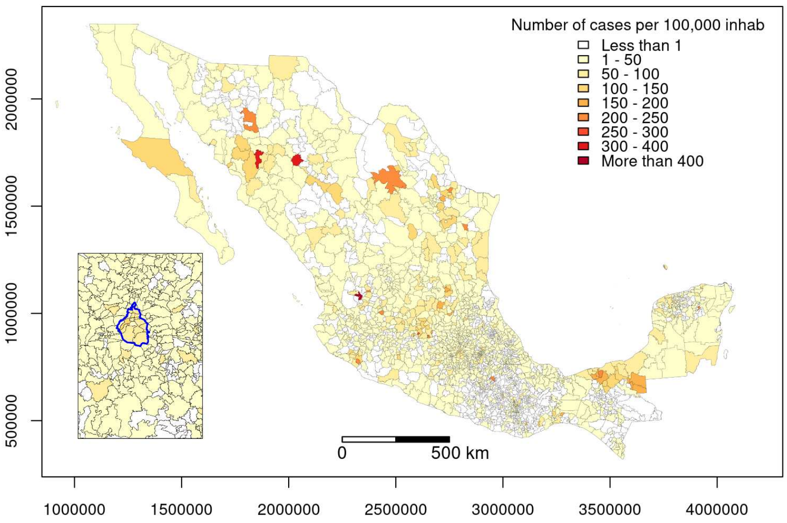

RR week 92 (2021-09-26 to 2021-10-02)

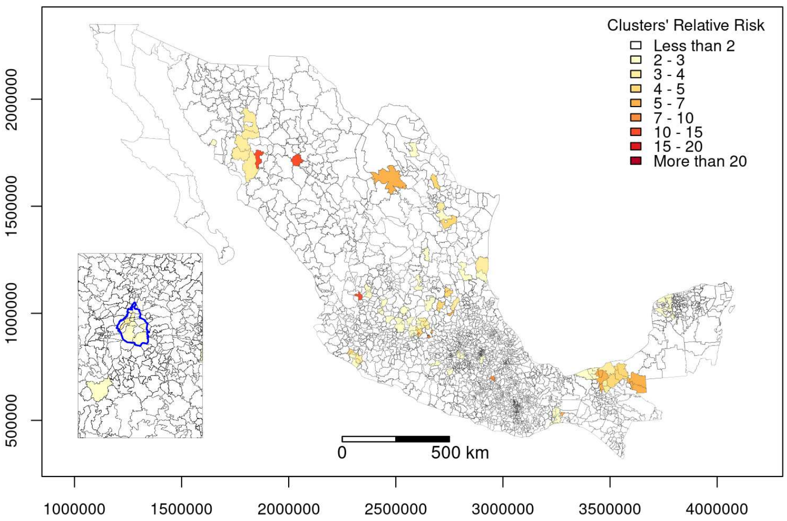

3 Effect of the different types of distance on neighbouring relationships

The following figures represent the n nearest neighbours (blue polygons) of Guanajuato's municipality (orange polygon) using Euclidean, least cost, resistance distances and the connectivity obtained by a gravitational model.

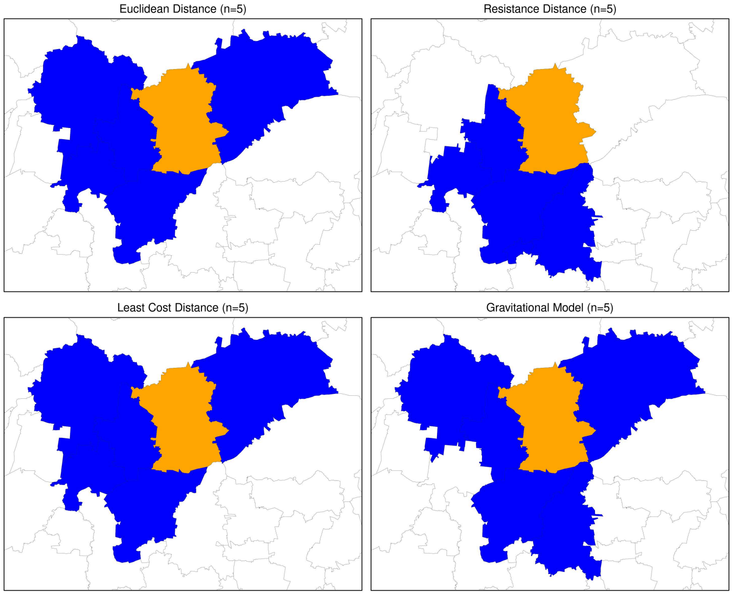

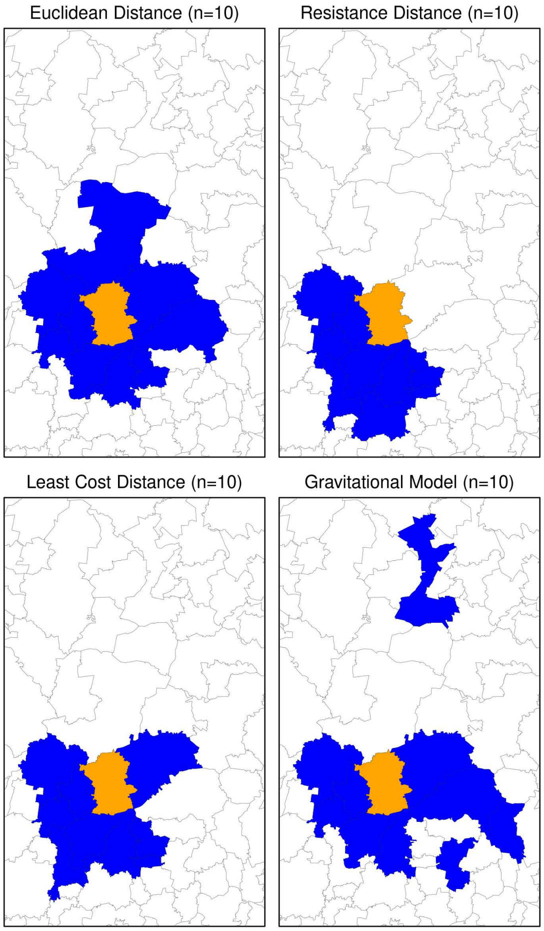
Euclidean Distance (n=10)
Resistance Distance (n=10)
Least Cost Distance (n=10)
Gravitational Model (n=10)

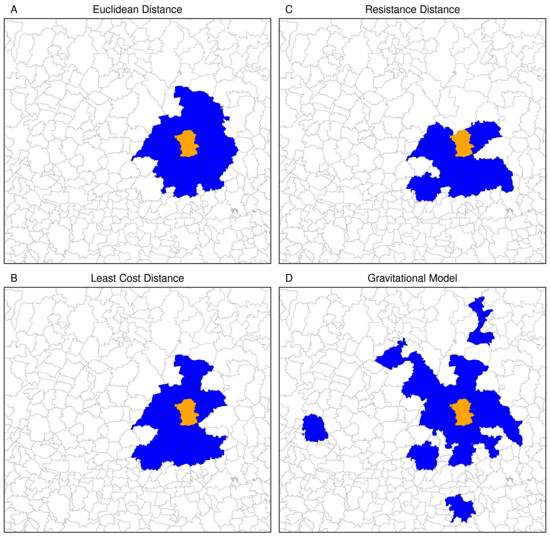
A
Euclidean Distance
C
Resistance Distance
B
Least Cost Distance
D
Gravitational Model

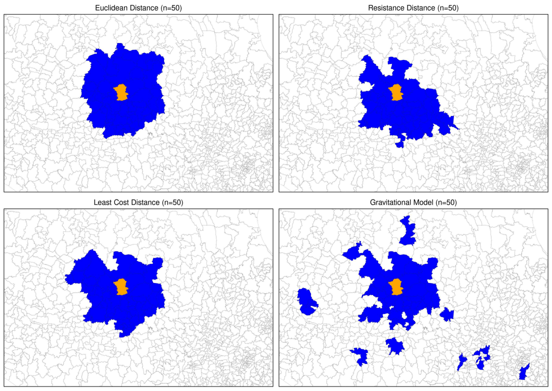
Euclidean Distance (n=50)
Resistance Distance (n=50)
Least Cost Distance (n=50)
Gravitational Model (n=50)

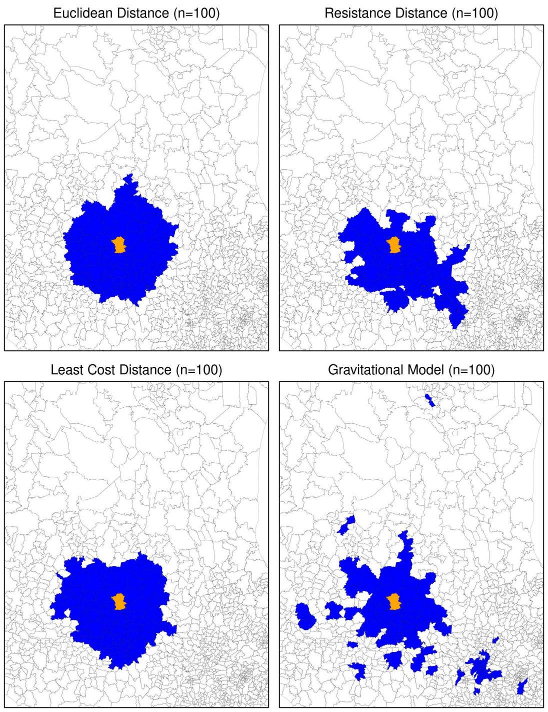
Euclidean Distance (n=100)
Resistance Distance (n=100)
Least Cost Distance (n=100)
Gravitational Model (n=100)

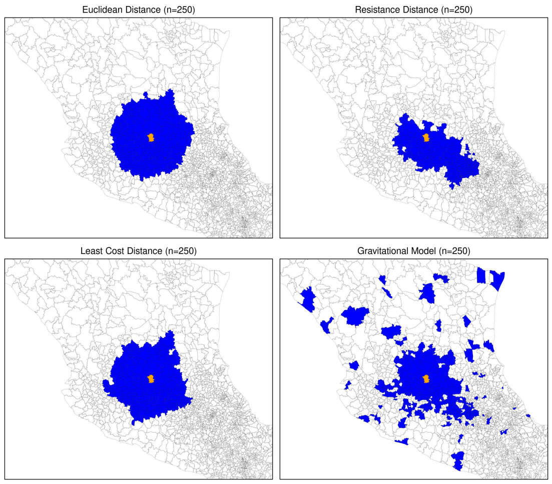
Euclidean Distance (n=250)
Resistance Distance (n=250)
Least Cost Distance (n=250)
Gravitational Model (n=250)

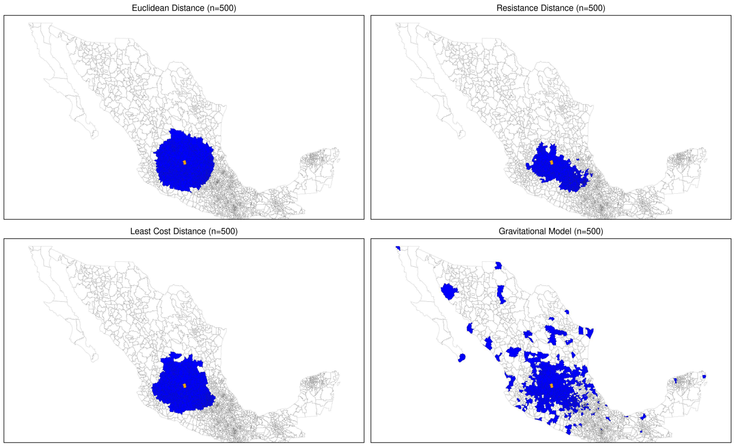
Euclidean Distance (n=500)
Resistance Distance (n=500)
Least Cost Distance (n=500)
Gravitational Model (n=500)

4 AUCs obtained by the different types of distance

The mean AUC was computed taking into account a different number of nearest neighbours N (N = 5, 10, 50, 100, 250 and 500).

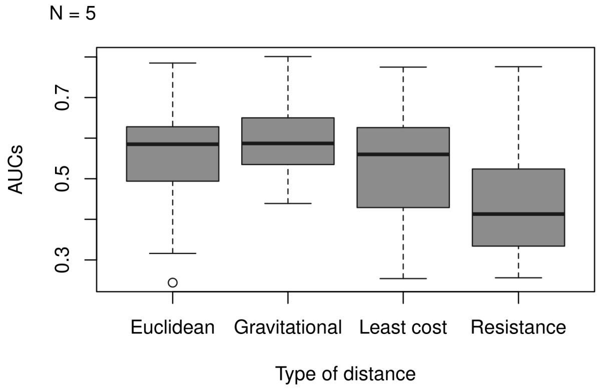

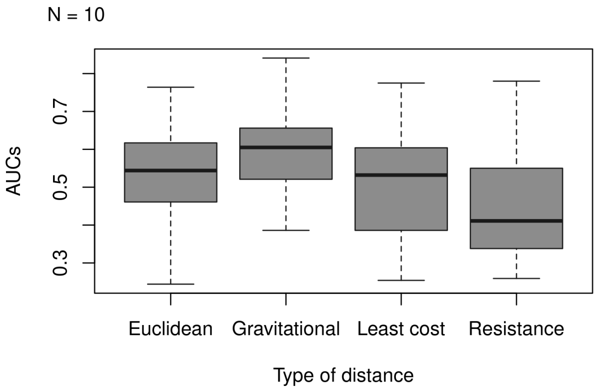
N = 10
AUCs
0.3
0.5
0.7
Euclidean
Gravitational
Least cost
Resistance
Type of distance

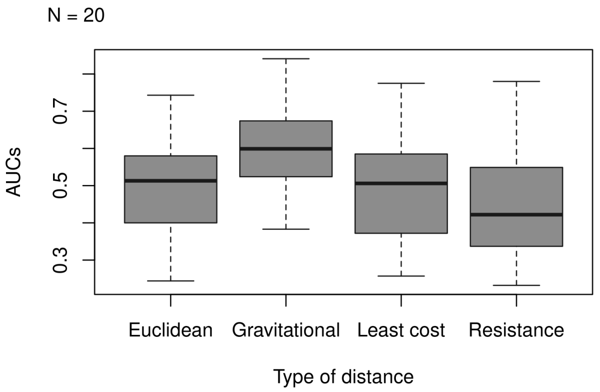
N = 20
AUCs
0.3
0.5
0.7
Euclidean
Gravitational
Least cost
Resistance
Type of distance

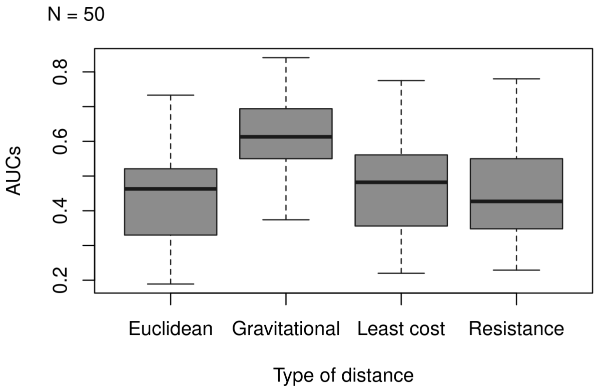
N = 50
AUCs
0.2
0.4
0.6
0.8
Euclidean
Gravitational
Least cost
Resistance
Type of distance

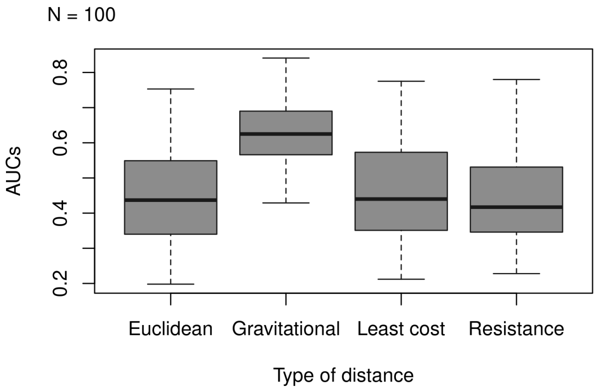
N = 100
AUCs
0.2
0.4
0.6
0.8
Euclidean
Gravitational
Least cost
Resistance
Type of distance

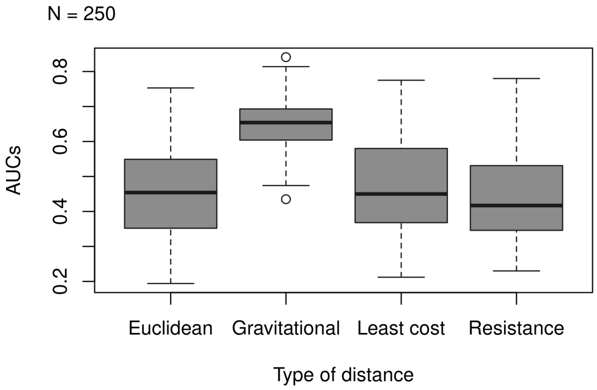
N = 250
AUCs
0.2
0.4
0.6
0.8
Euclidean
Gravitational
Least cost
Resistance
Type of distance

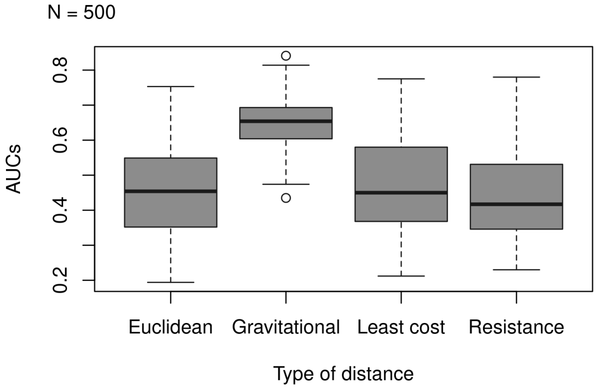
N = 500
AUCs
0.2
0.4
0.6
0.8
Euclidean
Gravitational
Least cost
Resistance
Type of distance

5 AUCs obtained by the different types of distance over time

The mean weekly AUC was computed taking into account a different number of nearest neighbours N (N = 5, 10, 20, 50, 100, 250 and 500).

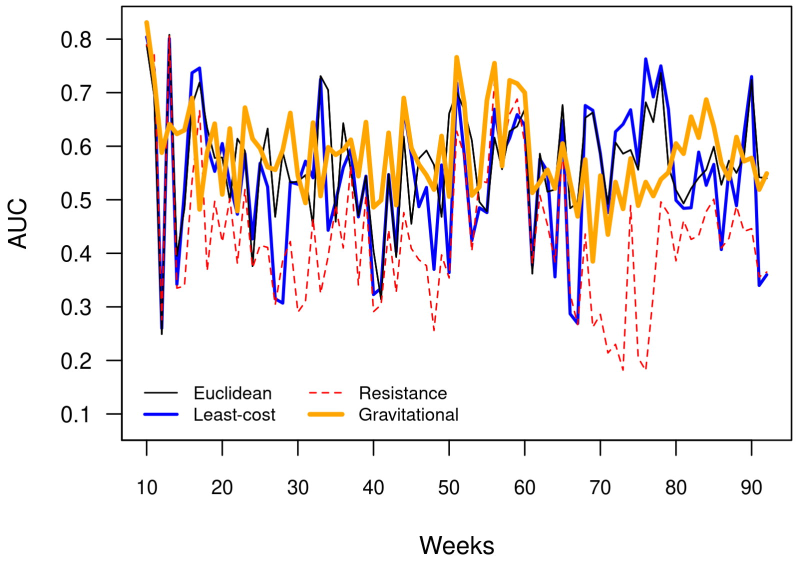

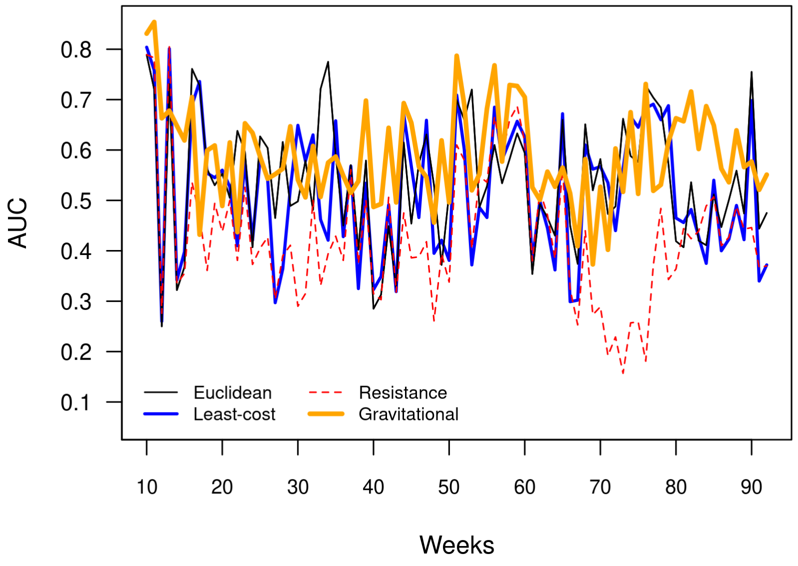
AUC
Weeks
0.1
0.2
0.3
0.4
0.5
0.6
0.7
0.8
10
20
30
40
50
60
70
80
90
Euclidean
Least-cost
Resistance
Gravitational

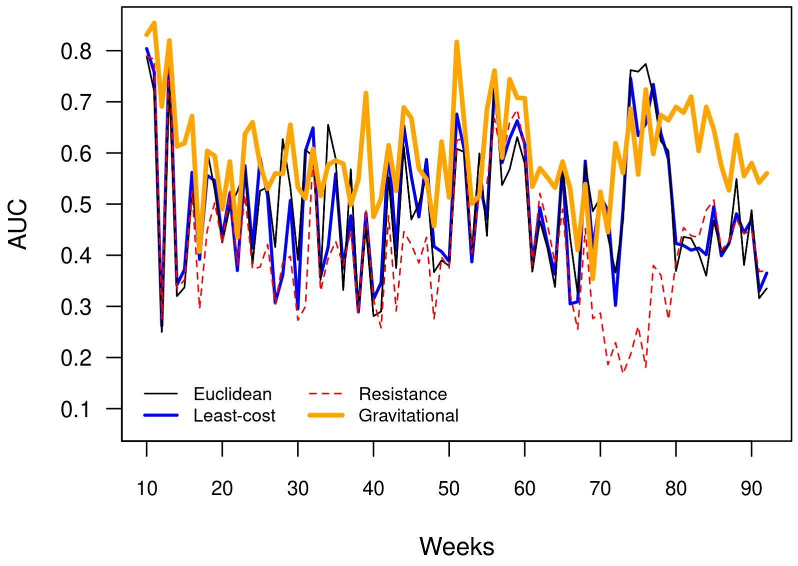
AUC
Weeks
0.1
0.2
0.3
0.4
0.5
0.6
0.7
0.8
10
20
30
40
50
60
70
80
90
Euclidean
Least-cost
Resistance
Gravitational

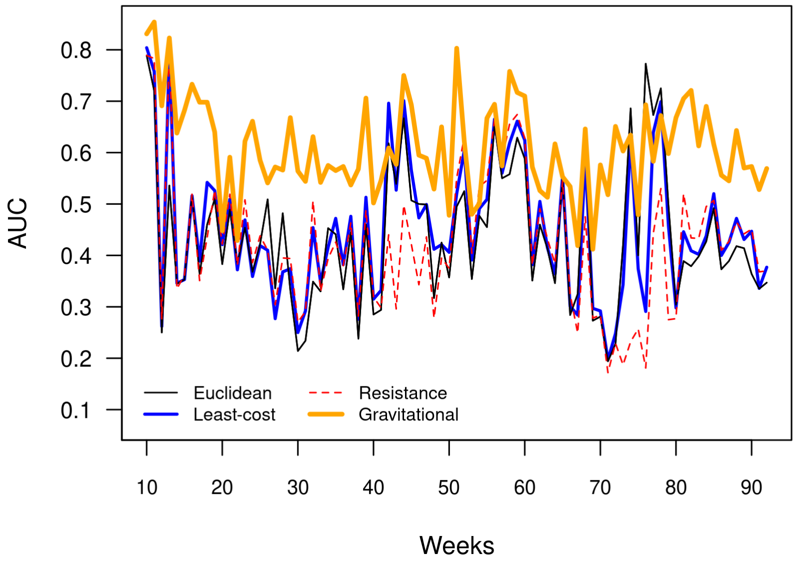
AUC
0.8
0.7
0.6
0.5
0.4
0.3
0.2
0.1
Euclidean
Least-cost
Resistance
Gravitational
10
20
30
40
50
60
70
80
90
Weeks

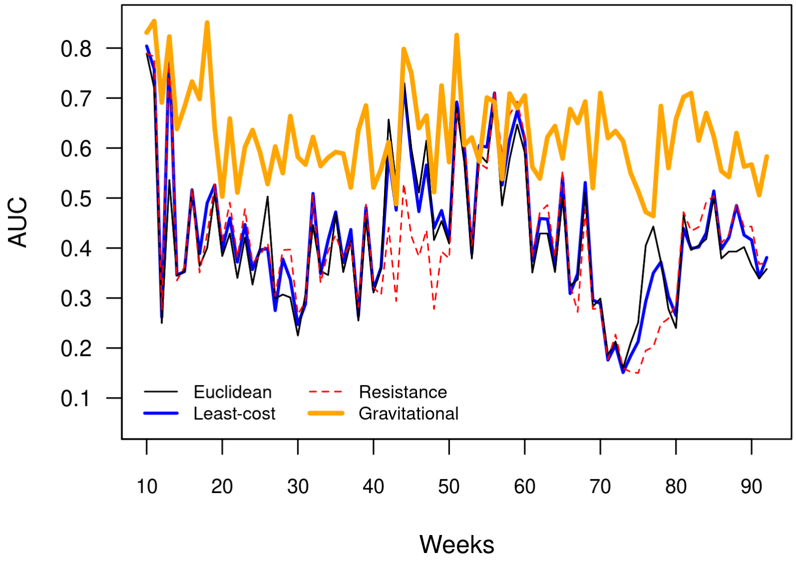
AUC
0.8
0.7
0.6
0.5
0.4
0.3
0.2
0.1
Euclidean
Least-cost
Resistance
Gravitational
10
20
30
40
50
60
70
80
90
Weeks

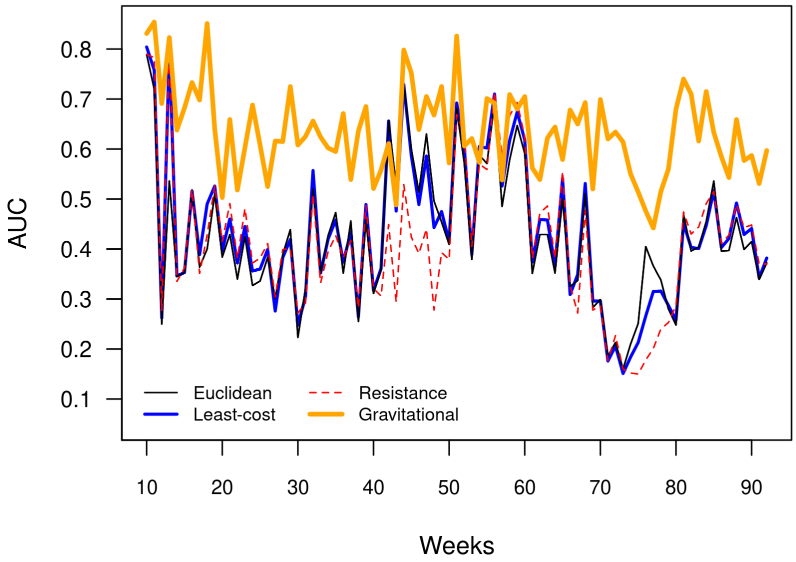
AUC
0.1
0.2
0.3
0.4
0.5
0.6
0.7
0.8
10
20
30
40
50
60
70
80
90
Weeks
Euclidean
Least-cost
Resistance
Gravitational

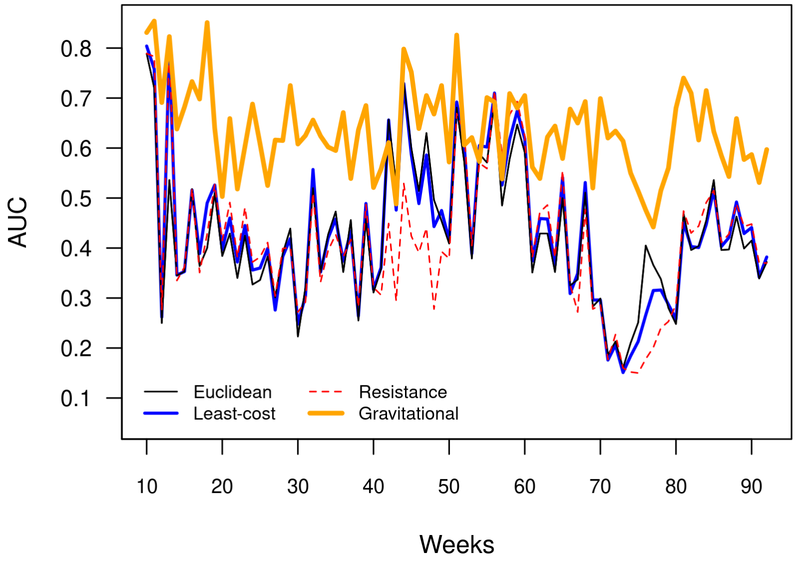
AUC
0.1
0.2
0.3
0.4
0.5
0.6
0.7
0.8
10
20
30
40
50
60
70
80
90
Weeks
Euclidean
Least-cost
Resistance
Gravitational

6 Equivalence between week number and dates

	**Week number**	**From**	**To**
1	9	2020-02-23	2020-02-29
2	10	2020-03-01	2020-03-07
3	11	2020-03-08	2020-03-14
4	12	2020-03-15	2020-03-21
5	13	2020-03-22	2020-03-28
6	14	2020-03-29	2020-04-04
7	15	2020-04-05	2020-04-11
8	16	2020-04-12	2020-04-18
9	17	2020-04-19	2020-04-25
10	18	2020-04-26	2020-05-02
11	19	2020-05-03	2020-05-09
12	20	2020-05-10	2020-05-16
13	21	2020-05-17	2020-05-23
14	22	2020-05-24	2020-05-30
15	23	2020-05-31	2020-06-06
16	24	2020-06-07	2020-06-13
17	25	2020-06-14	2020-06-20
18	26	2020-06-21	2020-06-27
19	27	2020-06-28	2020-07-04
20	28	2020-07-05	2020-07-11
21	29	2020-07-12	2020-07-18
22	30	2020-07-19	2020-07-25
23	31	2020-07-26	2020-08-01
24	32	2020-08-02	2020-08-08
25	33	2020-08-09	2020-08-15
26	34	2020-08-16	2020-08-22

	Week number	From	To
27	35	2020-08-23	2020-08-29
28	36	2020-08-30	2020-09-05
29	37	2020-09-06	2020-09-12
30	38	2020-09-13	2020-09-19
31	39	2020-09-20	2020-09-26
32	40	2020-09-27	2020-10-03
33	41	2020-10-04	2020-10-10
34	42	2020-10-11	2020-10-17
35	43	2020-10-18	2020-10-24
36	44	2020-10-25	2020-10-31
37	45	2020-11-01	2020-11-07
38	46	2020-11-08	2020-11-14
39	47	2020-11-15	2020-11-21
40	48	2020-11-22	2020-11-28
41	49	2020-11-29	2020-12-05
42	50	2020-12-06	2020-12-12
43	51	2020-12-13	2020-12-19
44	52	2020-12-20	2020-12-26
45	53	2020-12-27	2021-01-02
46	54	2021-01-03	2021-01-09
47	55	2021-01-10	2021-01-16
48	56	2021-01-17	2021-01-23
49	57	2021-01-24	2021-01-30
50	58	2021-01-31	2021-02-06
51	59	2021-02-07	2021-02-13
52	60	2021-02-14	2021-02-20
53	61	2021-02-21	2021-02-27
54	62	2021-02-28	2021-03-06
55	63	2021-03-07	2021-03-13
56	64	2021-03-14	2021-03-20
57	65	2021-03-21	2021-03-27
58	66	2021-03-28	2021-04-03
59	67	2021-04-04	2021-04-10
60	68	2021-04-11	2021-04-17
61	69	2021-04-18	2021-04-24
62	70	2021-04-25	2021-05-01

	Week number	From	To
63	71	2021-05-02	2021-05-08
64	72	2021-05-09	2021-05-15
65	73	2021-05-16	2021-05-22
66	74	2021-05-23	2021-05-29
67	75	2021-05-30	2021-06-05
68	76	2021-06-06	2021-06-12
69	77	2021-06-13	2021-06-19
70	78	2021-06-20	2021-06-26
71	79	2021-06-27	2021-07-03
72	80	2021-07-04	2021-07-10
73	81	2021-07-11	2021-07-17
74	82	2021-07-18	2021-07-24
75	83	2021-07-25	2021-07-31
76	84	2021-08-01	2021-08-07
77	85	2021-08-08	2021-08-14
78	86	2021-08-15	2021-08-21
79	87	2021-08-22	2021-08-28
80	88	2021-08-29	2021-09-04
81	89	2021-09-05	2021-09-11
82	90	2021-09-12	2021-09-18
83	91	2021-09-19	2021-09-25
84	92	2021-09-26	2021-10-02
